# Supplementary material for: The cnidarian parasite Ceratonova shasta utilizes inherited and recruited venom-like compounds during infection
Source: PeerJ. 2021 Dec 15;9:e12606. doi: 10.7717/peerj.12606 (PMC8684318; doi:10.7717/peerj.12606)

SeqID: TRINITY_DN59953_c0_g1_i1
Annotation: uncharacterized protein
Identified by seq analysis: N

Key: (X%; Y%) A / B X = Alignment coverage Y= %ID between query and temple A = Molecule name B = Molecule description
NP= Alignment & %ID not provided by Phyre2; Green = same clade; Purple = Earlier common ancestor; Red = Non-valid entry; Blue = Not in tree

3T5O Chain A (Alignment coverage%; identity%) Complement Component C6/ Perforin domain of MAC

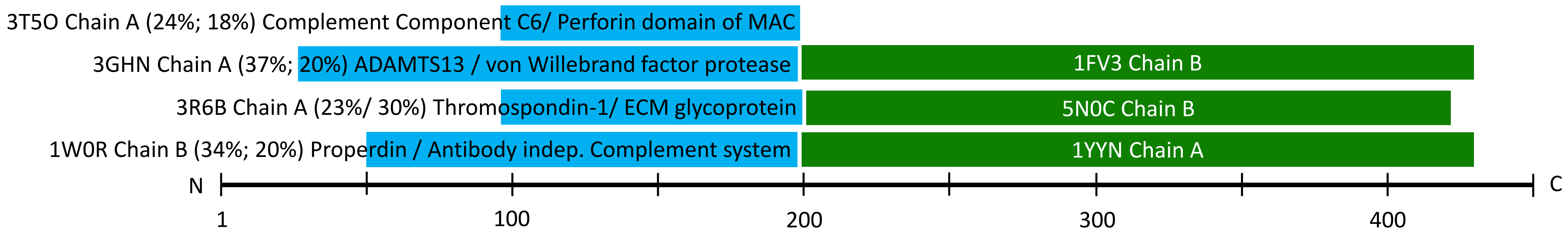

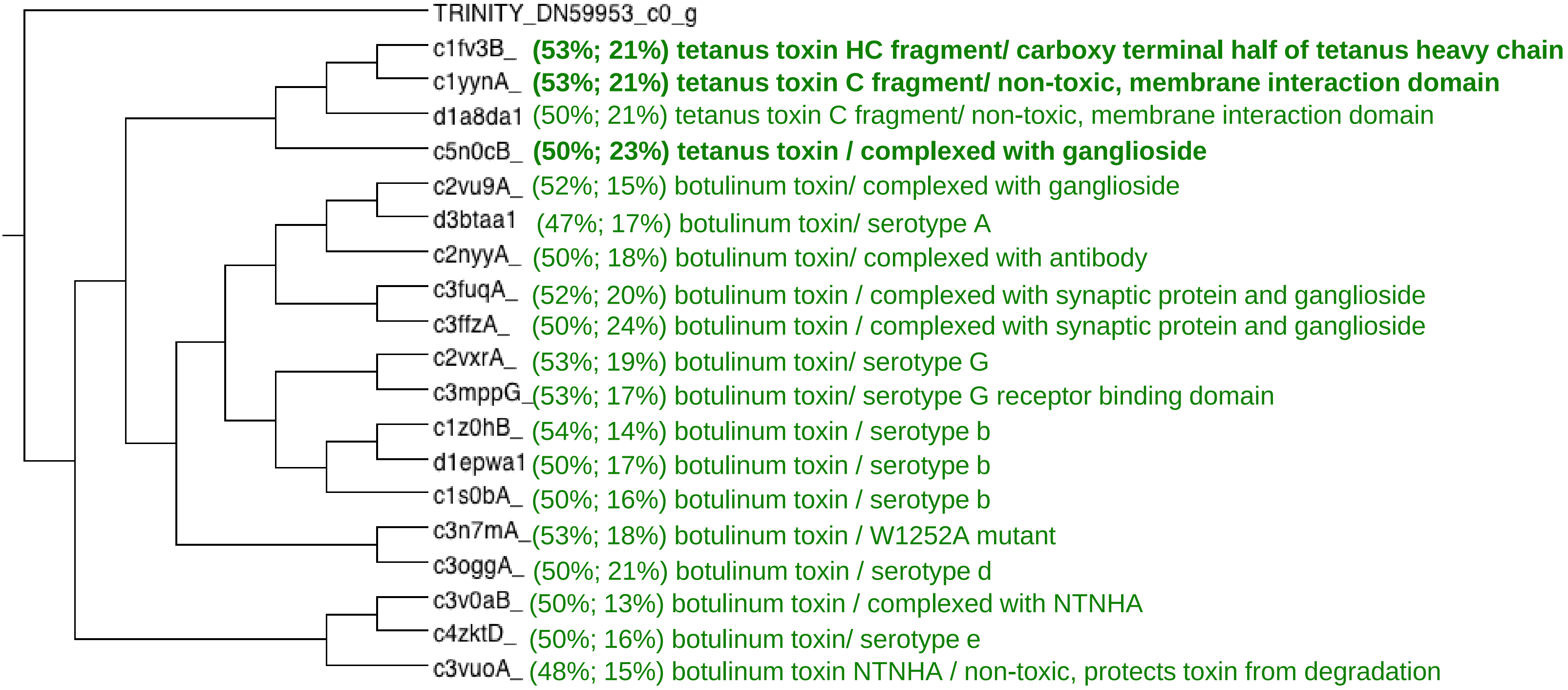

SeqID: TRINITY_DN9306_c0_g1_i1
Annotation: Nematobalst-specific nb012a
Identified by seq analysis: N

Key: (X%; Y%) A / B X = Alignment coverage Y= %ID between query and temple A = Molecule name B = Molecule description
NP= Alignment & %ID not provided by Phyre2; Green = same clade; Purple = Earlier common ancestor; Red = Non-valid entry; Blue = Not in tree

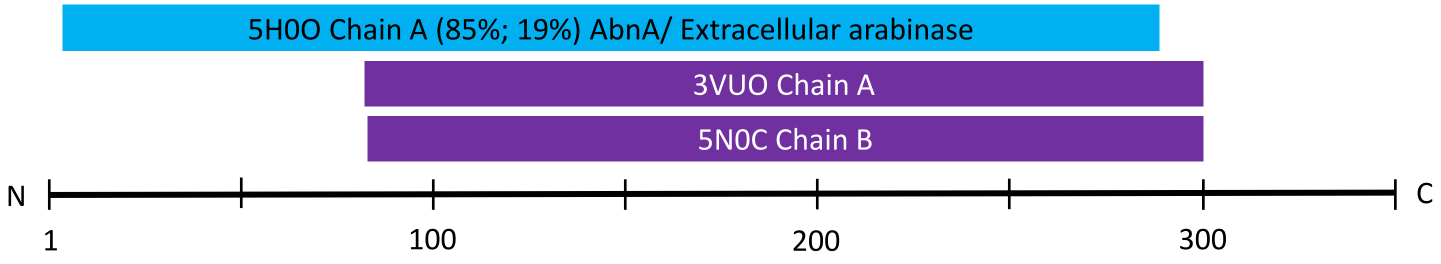

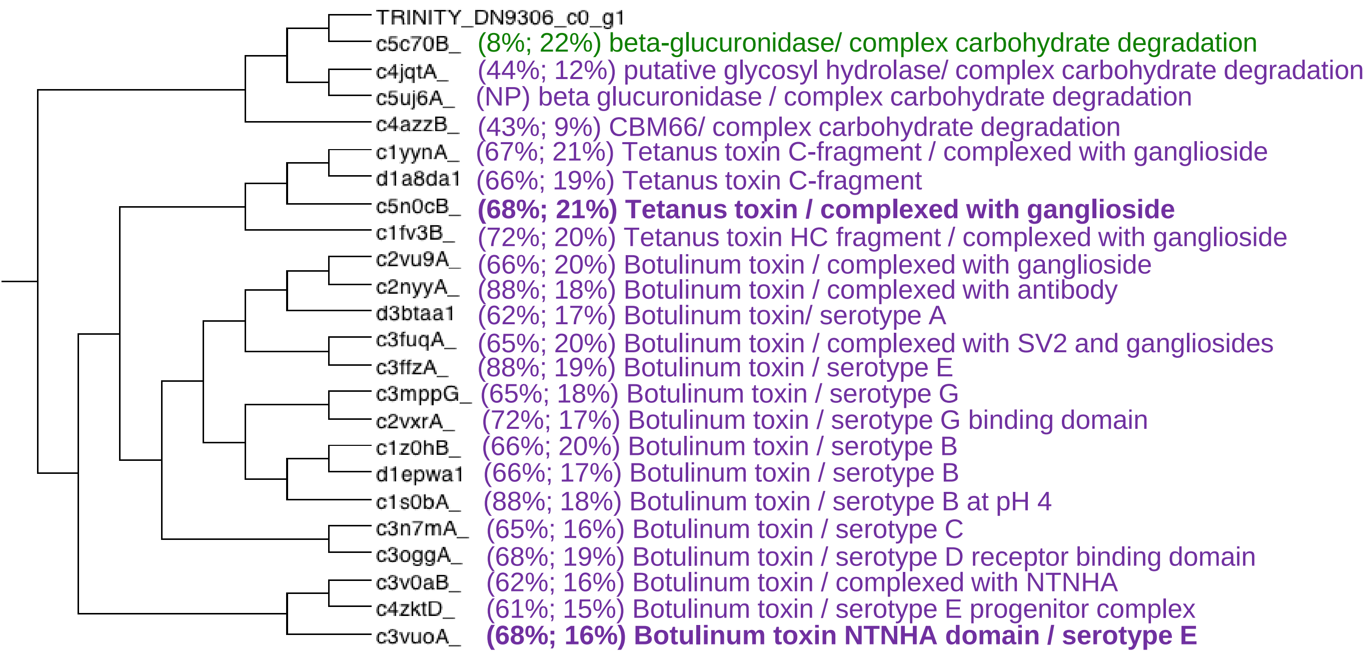

SeqID: TRINITY_DN60481_c0_g1_i1
Annotation: Nematogalectin C
Identified by seq analysis: Y

Key: (X%; Y%) A / B X = Alignment coverage Y= %ID between query and temple A = Molecule name B = Molecule description
NP= Alignment & %ID not provided by Phyre2; Yellow = Sequence from database; Green = same clade; Purple = Earlier common ancestor;
Red = Non-valid entry; Blue = Not in tree

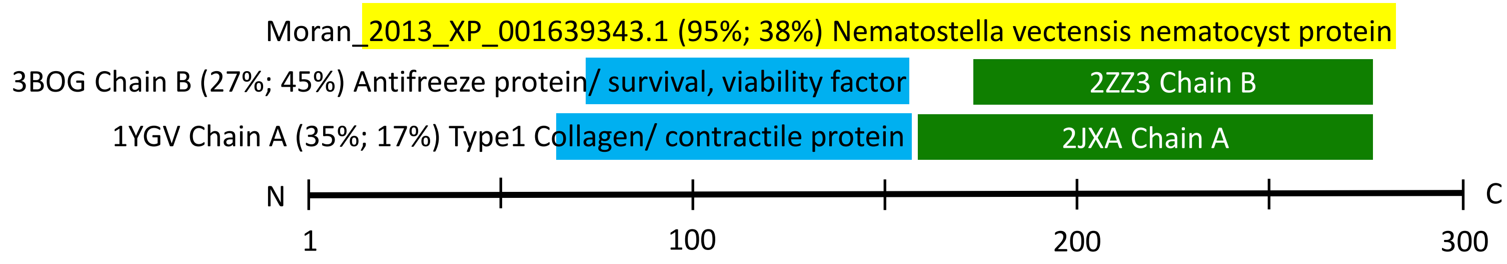

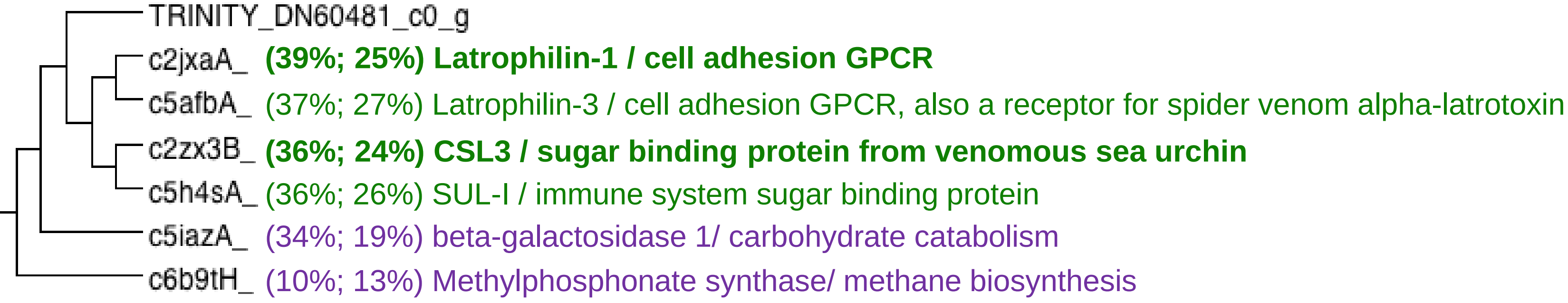

SeqID: TRINITY_DN60393_c0_g1_i1
Annotation: Nematogalectin-related
Identified by seq analysis: Y

Key: (X%; Y%) A / B X = Alignment coverage Y= %ID between query and temple A = Molecule name B = Molecule description
NP= Alignment & %ID not provided by Phyre2; Yellow = Sequence from database; Green = same clade; Purple = Earlier common ancestor; Red = Non-valid entry; Blue = Not in tree

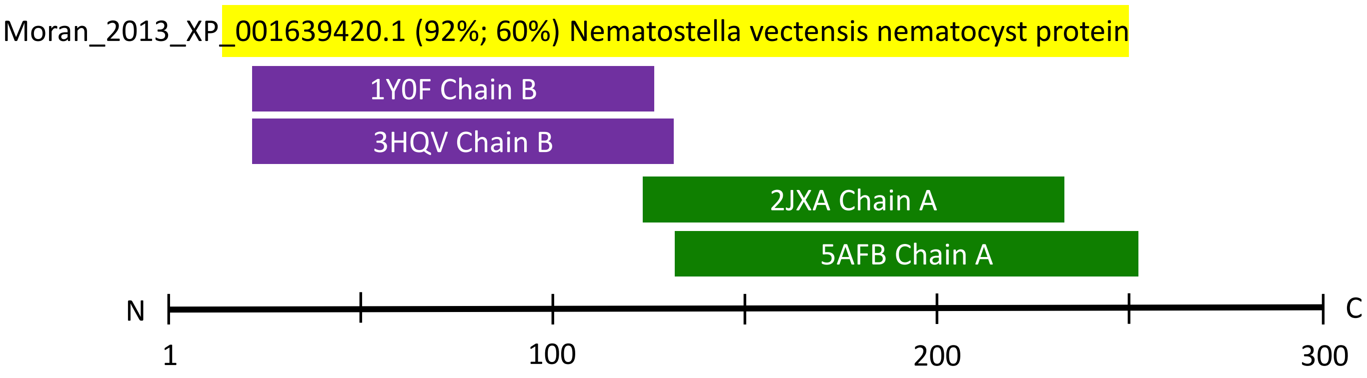

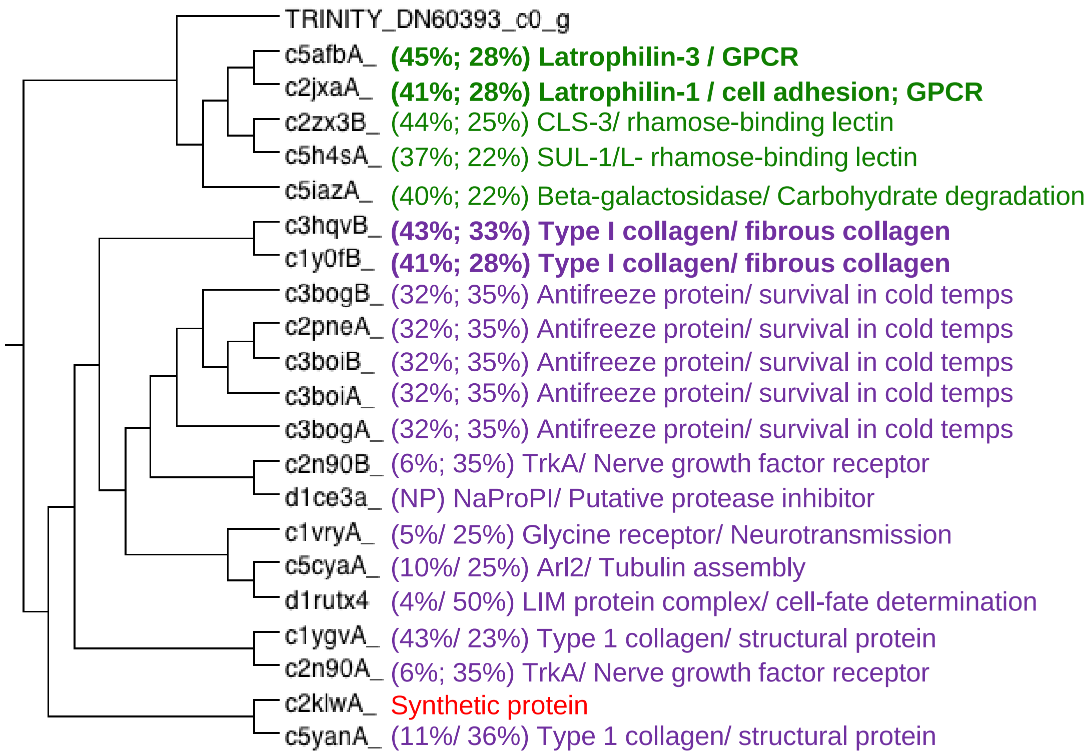

SeqID: TRINITY_DN4616_c0_g1_i1
Annotation: Macrophage mannose receptor 1-like
Identified by seq analysis: Y

Key: (X%; Y%) A / B X = Alignment coverage Y= %ID between query and temple A = Molecule name B = Molecule description
NP= Alignment & %ID not provided by Phyre2; Yellow = Sequence from database ; Green = same clade; Purple = Earlier common ancestor; Red = Non-valid entry; Blue = Not in tree

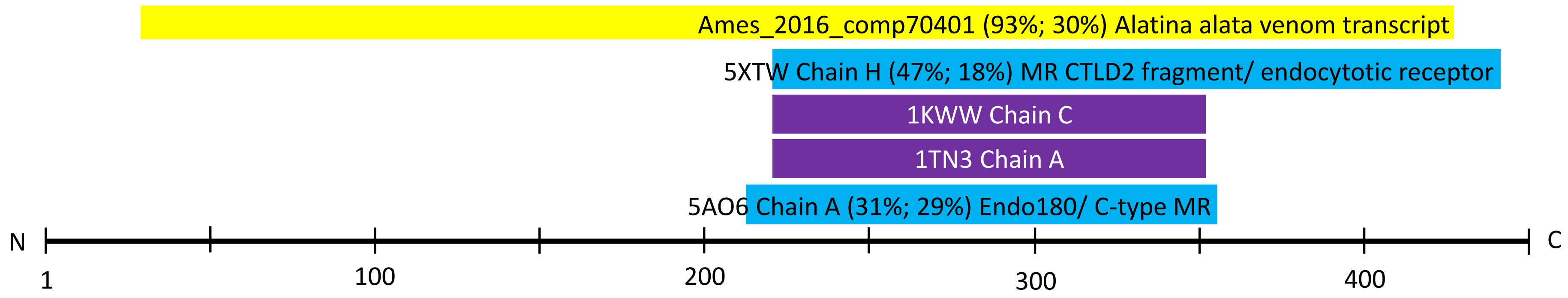

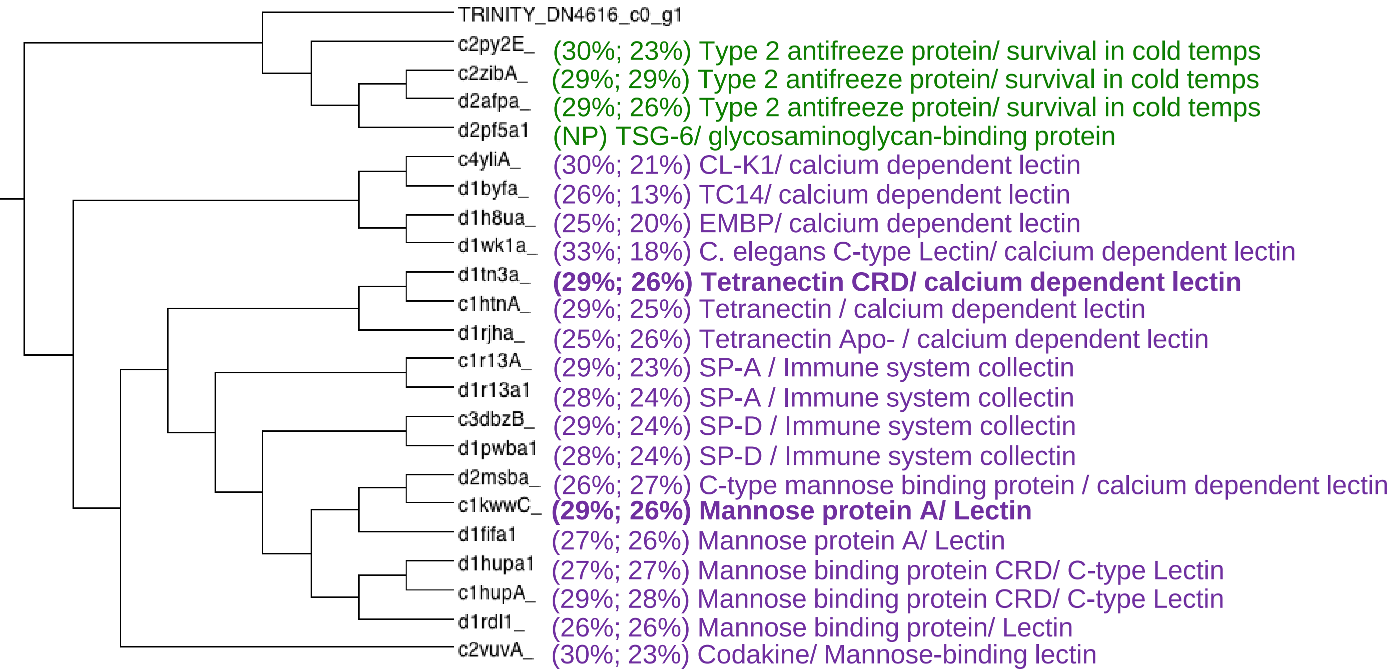

SeqID: TRINITY_DN60348_c0_g1_i1
Annotation: Nematogalectin A
Identified by seq analysis: Y

Key: (X%; Y%) A / B   X = Alignment coverage   Y= %ID between query and temple   A = Molecule name   B = Molecule description
NP= Alignment & %ID not provided by Phyre2; Yellow = Sequence from database ; Green = same clade; Purple = Earlier common ancestor; Red = Non-valid entry; Blue = Not in tree

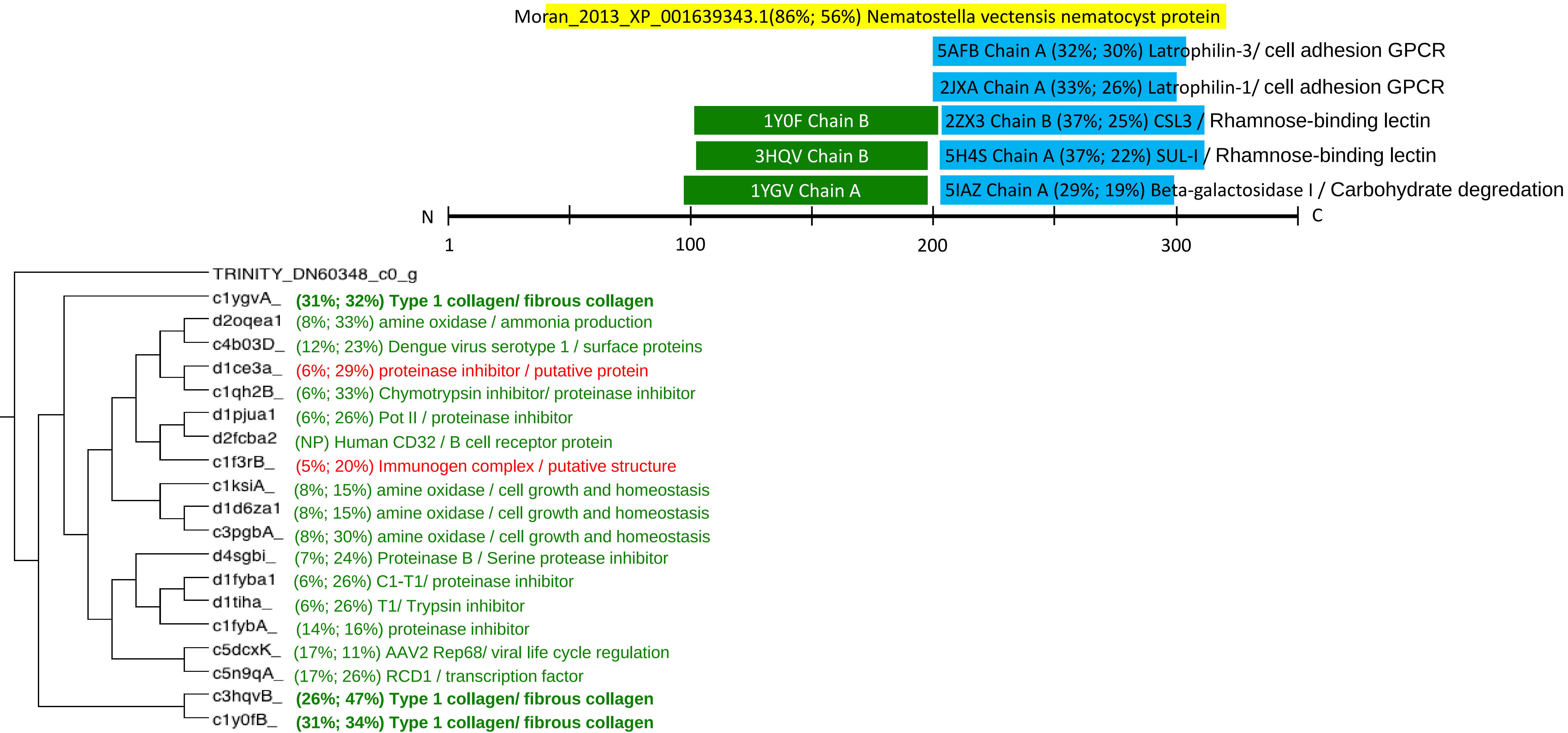

SeqID: TRINITY_DN34715_c0_g1_i1
Annotation: Adhesion G-coupled receptor B3-like
Identified by seq analysis: N

Key: (X%; Y%) A / B X = Alignment coverage Y= %ID between query and temple A = Molecule name B = Molecule description
NP= Alignment & %ID not provided by Phyre2; Green = same clade; Purple = Earlier common ancestor; Red = Non-valid entry; Blue = Not in tree

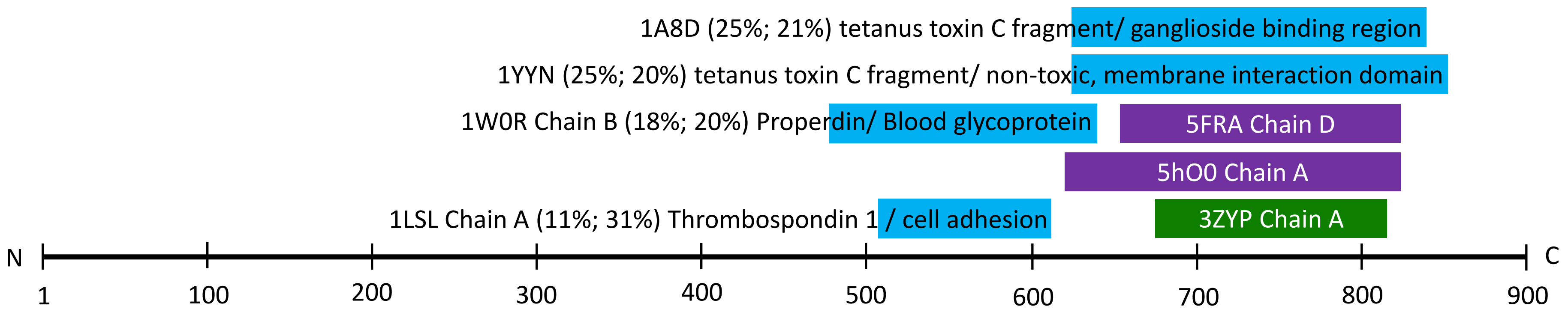

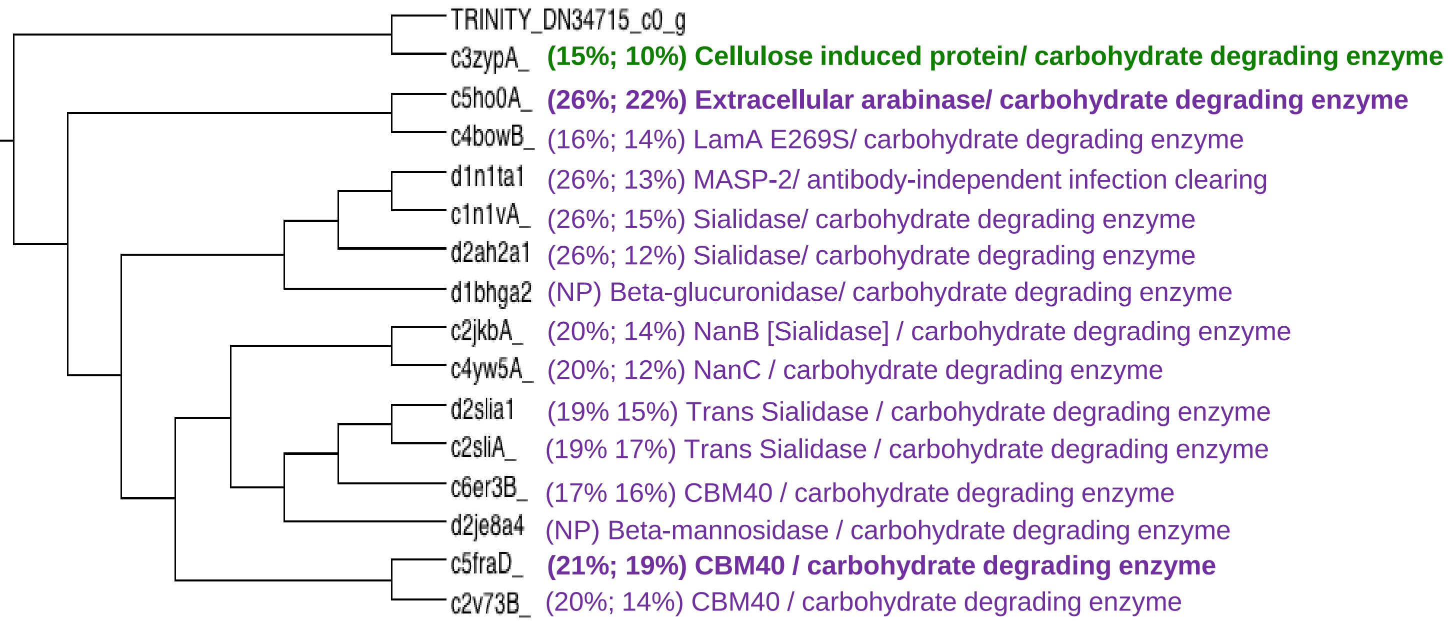

SeqID: TRINITY_DN6224_c0_g2_i1
Annotation: nematoblast-specific nb012a
Identified by seq analysis: Y

Key: (X%; Y%) A / B X = Alignment coverage Y= %ID between query and temple A = Molecule name B = Molecule description
NP= Alignment & %ID not provided by Phyre2; Yellow = Sequence from database ; Green = same clade; Purple = Earlier common ancestor; Red = Non-valid entry; Blue = Not in tree

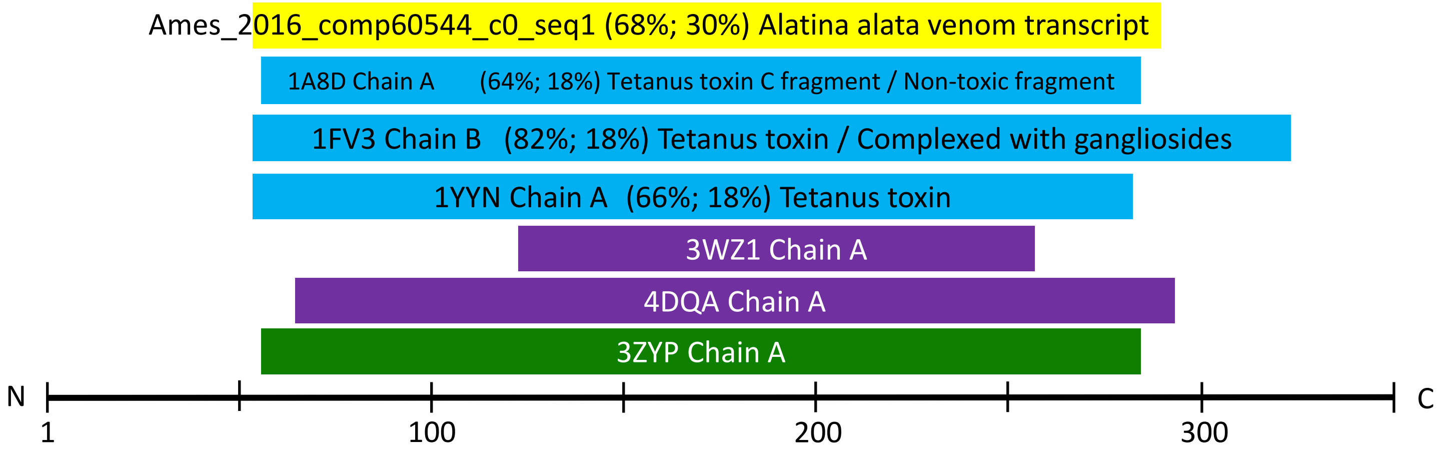

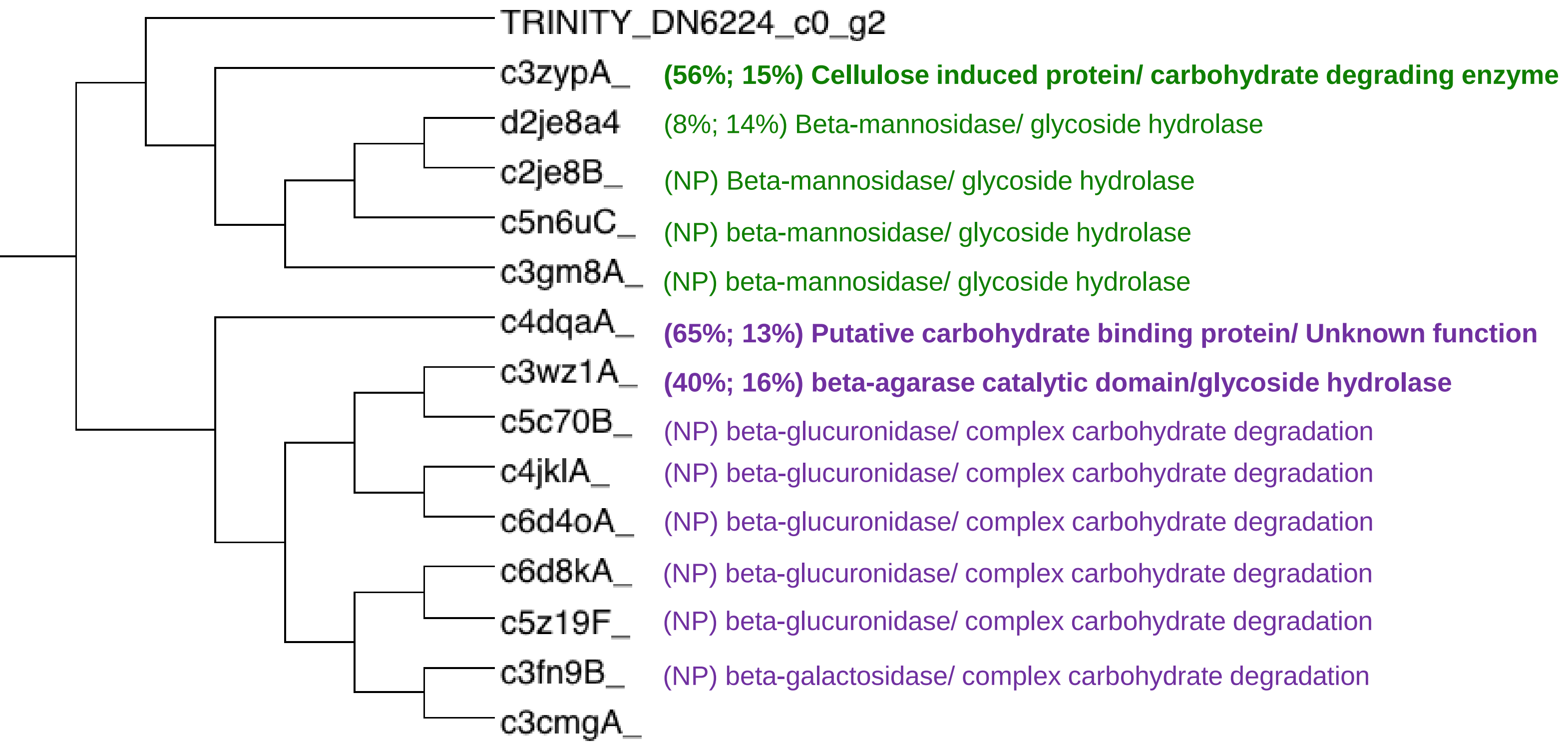

SeqID: TRINITY_DN782_c0_g1_i1
Annotation: Stefin 2
Identified by seq analysis: N

Key: (X%; Y%) A / B X = Alignment coverage Y= %ID between query and temple A = Molecule name B = Molecule description
NP= Alignment & %ID not provided by Phyre2; Green = same clade; Purple = Earlier common ancestor; Red = Non-valid entry; Blue = Not in tree

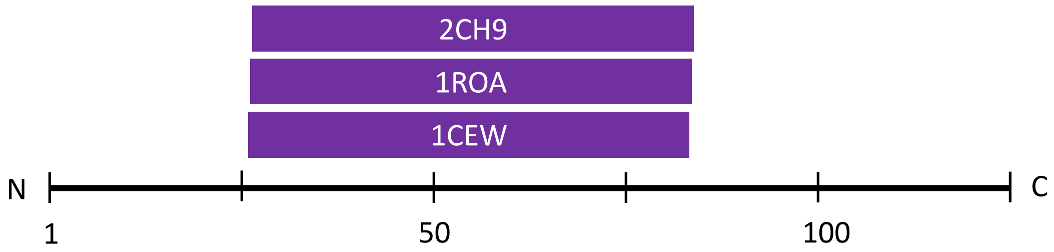

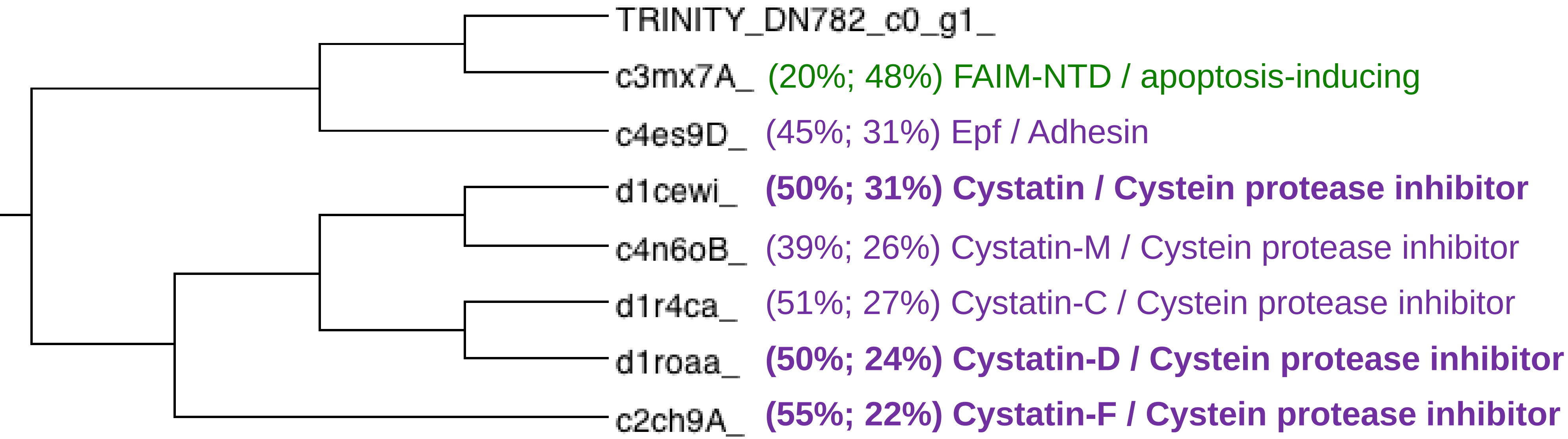

SeqID: TRINITY_DN55967_c1_g1_i2
Annotation: Peptidase inhibitor 16-like
Identified by seq analysis: Y

Key: (X%; Y%) A / B X = Alignment coverage Y= %ID between query and temple A = Molecule name B = Molecule description
NP= Alignment & %ID not provided by Phyre2; Yellow = Sequence from database; Green = same clade; Purple = Earlier common ancestor; Red = Non-valid entry; Blue = Not in tree

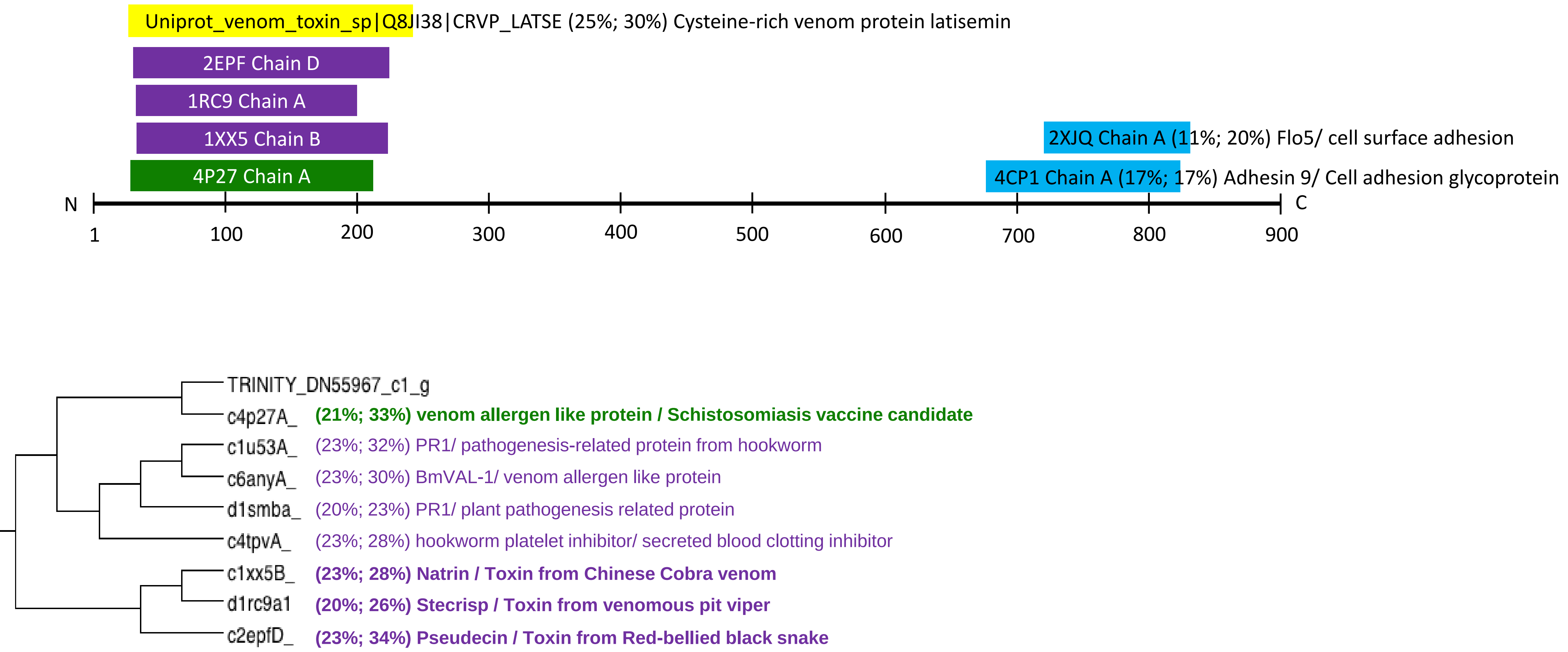

SeqID: TRINITY_DN47165_c0_g1_i4
Annotation: PC3-like endoprotease variant B
Identified by seq analysis: N

Key: (X%; Y%) A / B   X = Alignment coverage   Y= %ID between query and temple   A = Molecule name   B = Molecule description
NP= Alignment & %ID not provided by Phyre2; Green = same clade; Purple = Earlier common ancestor; Red = Non-valid entry; Blue = Not in tree

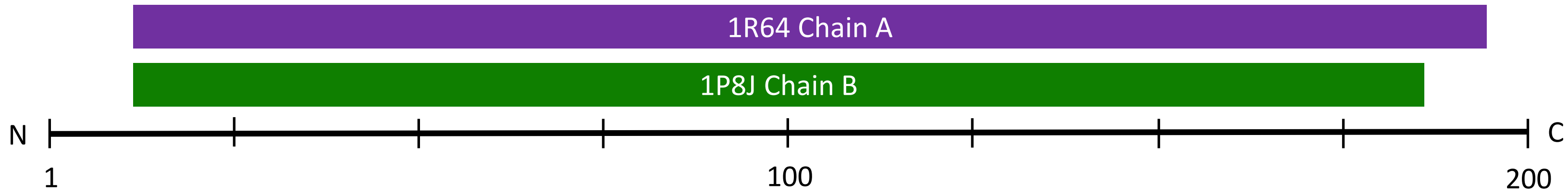

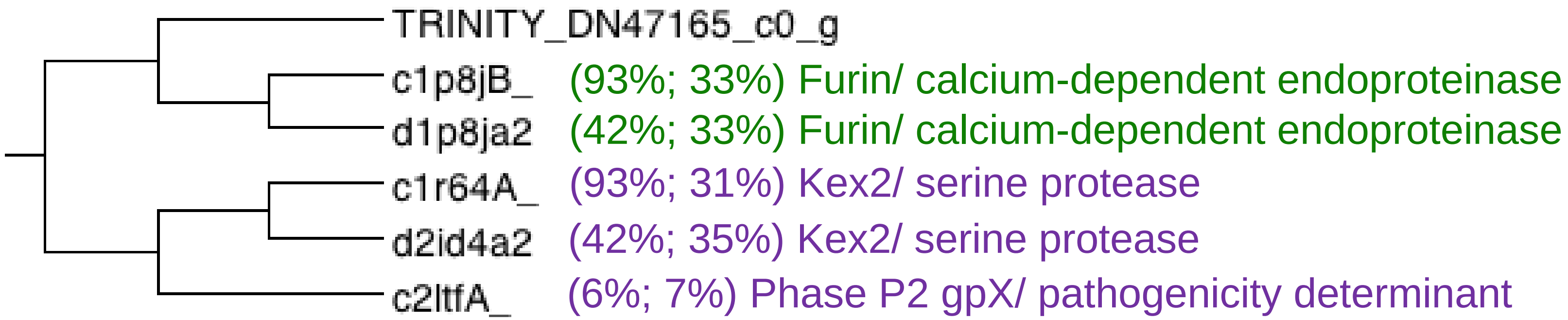

SeqID: TRINITY_DN48395_c1_g1_i1
Annotation: prostatic acid phosphatase isoform X2
Identified by seq analysis: Y

Key: (X%; Y%) A / B X = Alignment coverage Y= %ID between query and temple A = Molecule name B = Molecule description
NP= Alignment & %ID not provided by Phyre2; Yellow = Sequence from database; Green = same clade; Purple = Earlier common ancestor; Red = Non-valid entry; Blue = Not in tree

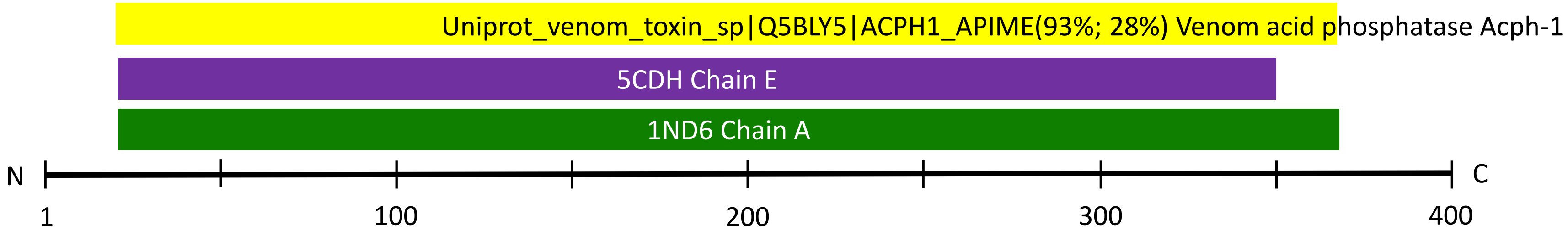

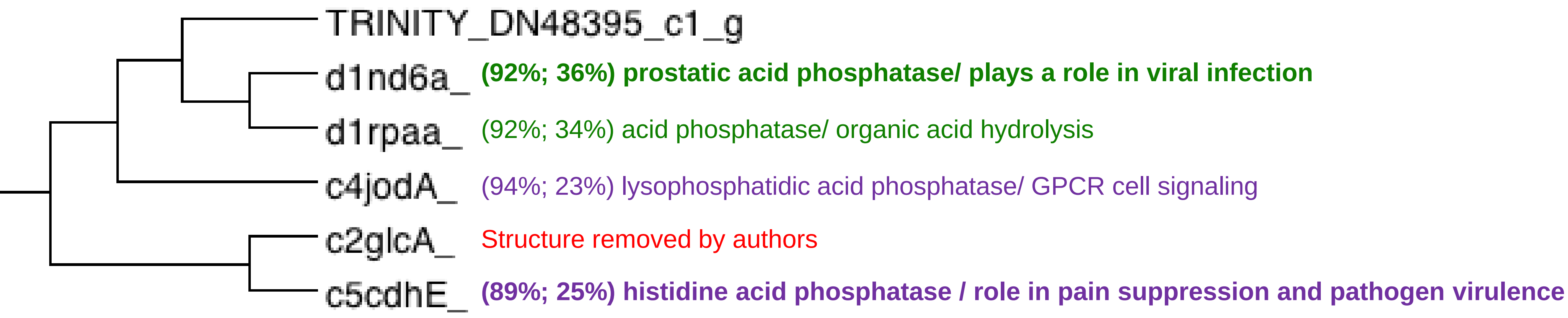

SeqID: TRINITY_DN47165_c0_g1_i5
Annotation: PC3-like endoprotease variant B
Identified by seq analysis: N

Key: (X%; Y%) A / B X = Alignment coverage Y= %ID between query and temple A = Molecule name B = Molecule description
NP= Alignment & %ID not provided by Phyre2; Green = same clade; Purple = Earlier common ancestor; Red = Non-valid entry; Blue = Not in tree

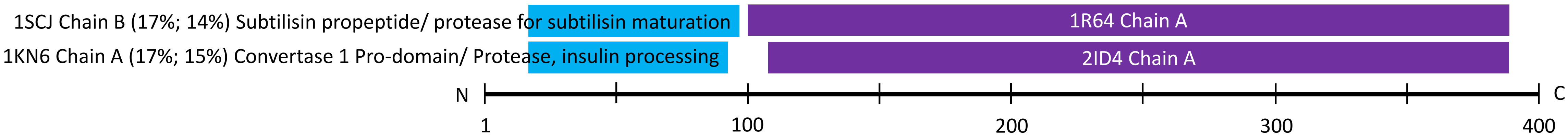

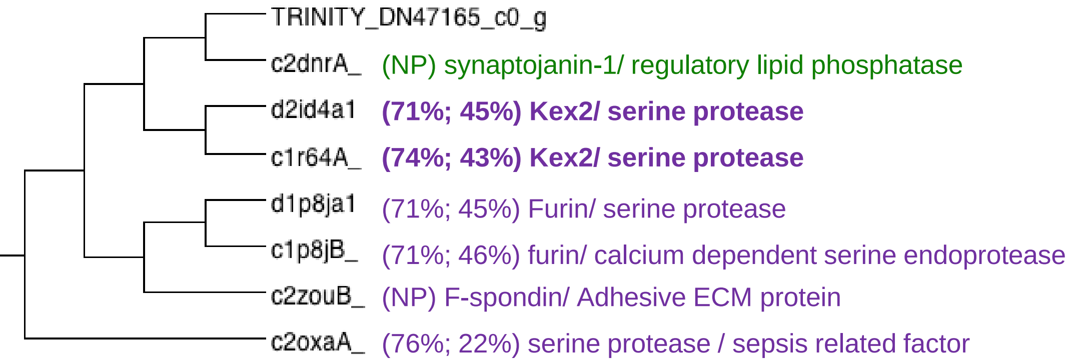

SeqID: TRINITY_DN57920_c0_g1_i1
Annotation: Carboxypeptidase N catalytic chain
Identified by seq analysis: Y

Key: (X%; Y%) A / B X = Alignment coverage Y= %ID between query and temple A = Molecule name B = Molecule description
NP= Alignment & %ID not provided by Phyre2; Yellow = Sequence from database; Green = same clade; Purple = Earlier common ancestor; Red = Non-valid entry; Blue = Not in tree

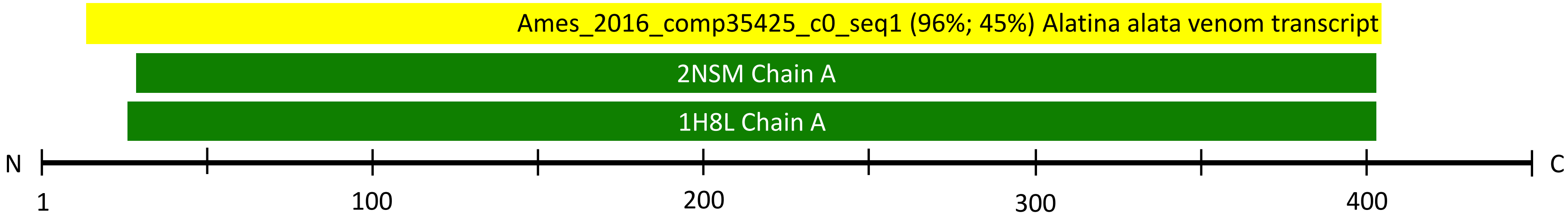

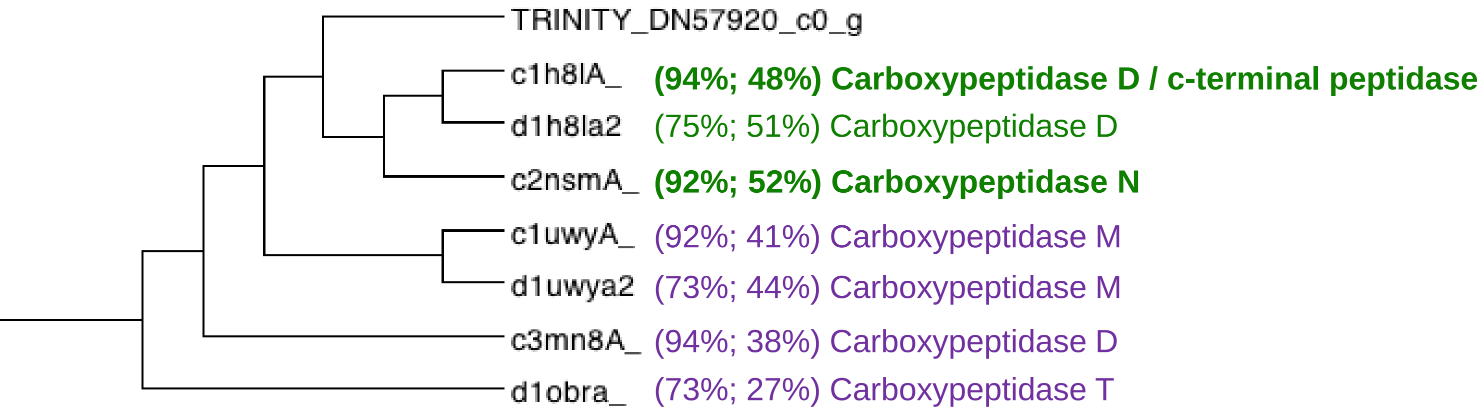

SeqID: TRINITY_DN54320_c0_g1_i1
Annotation: Carboxypeptidase_D
Identified by seq analysis: Y

Key: (X%; Y%) A / B X = Alignment coverage Y= %ID between query and temple A = Molecule name B = Molecule description
NP= Alignment & %ID not provided by Phyre2; Yellow = Sequence from database; Green = same clade; Purple = Earlier common ancestor; Red = Non-valid entry; Blue = Not in tree

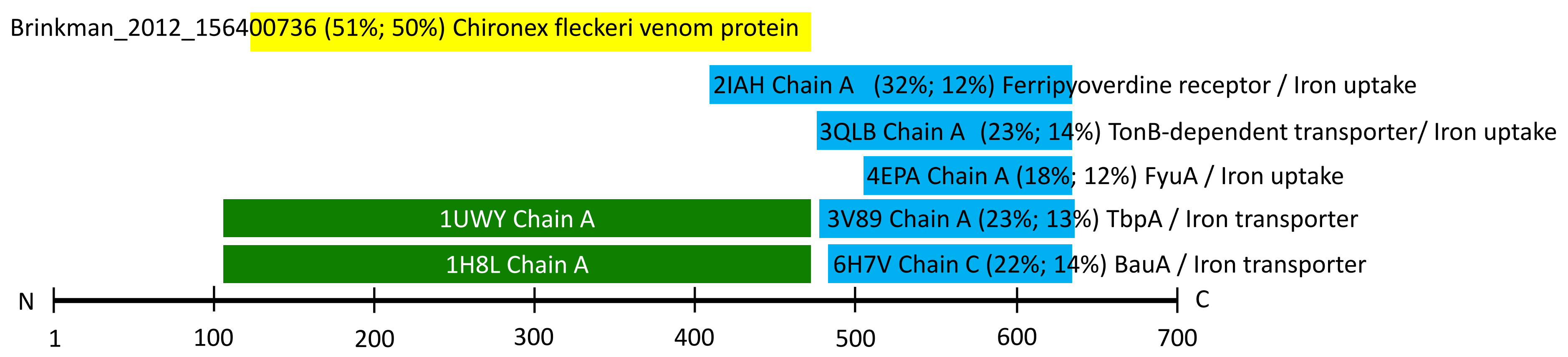

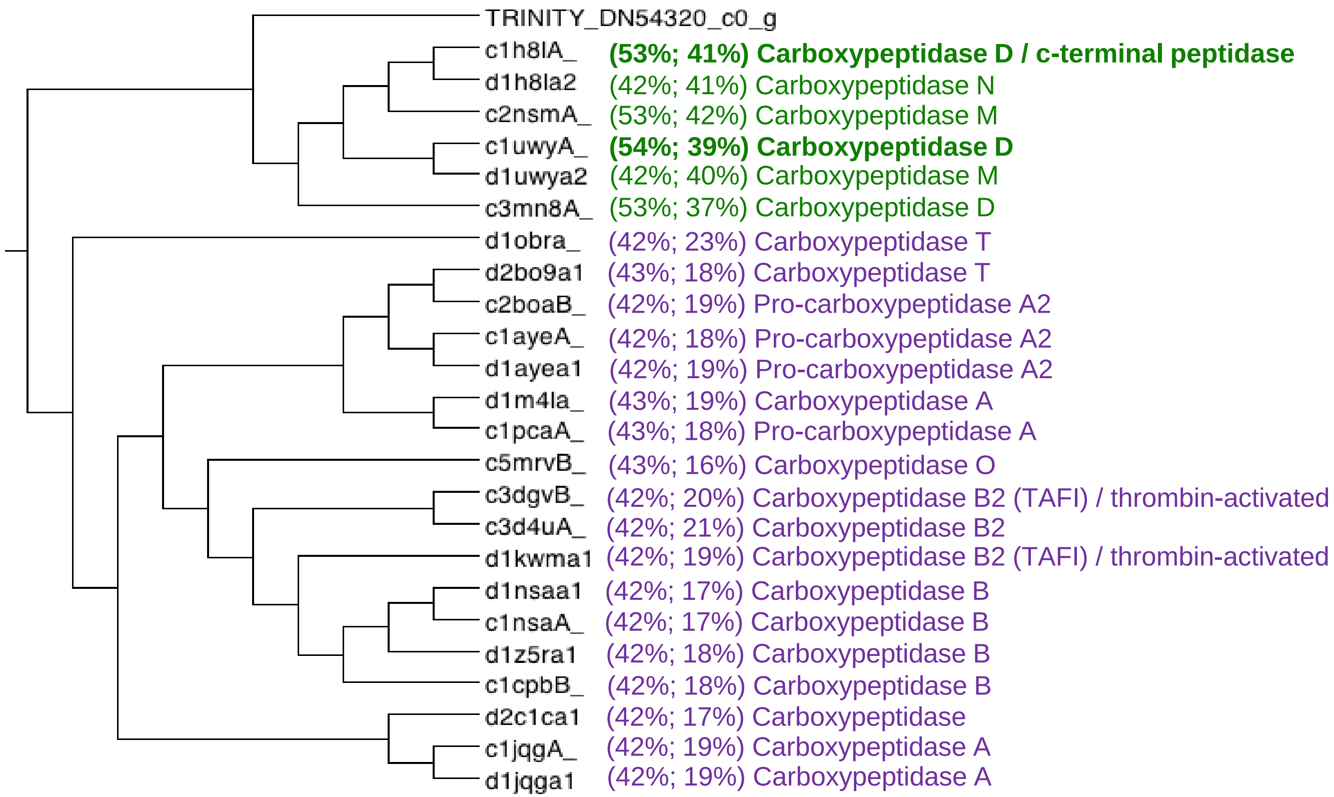

SeqID: TRINITY_DN40435_c0_g1_i1
Annotation: endoplasmic reticulum aminopeptidase 1-like
Identified by seq analysis: Y

Key: (X%; Y%) A / B X = Alignment coverage Y= %ID between query and temple A = Molecule name B = Molecule description
NP= Alignment & %ID not provided by Phyre2; Yellow = Sequence from database; Green = same clade; Purple = Earlier common ancestor; Red = Non-valid entry; Blue = Not in tree

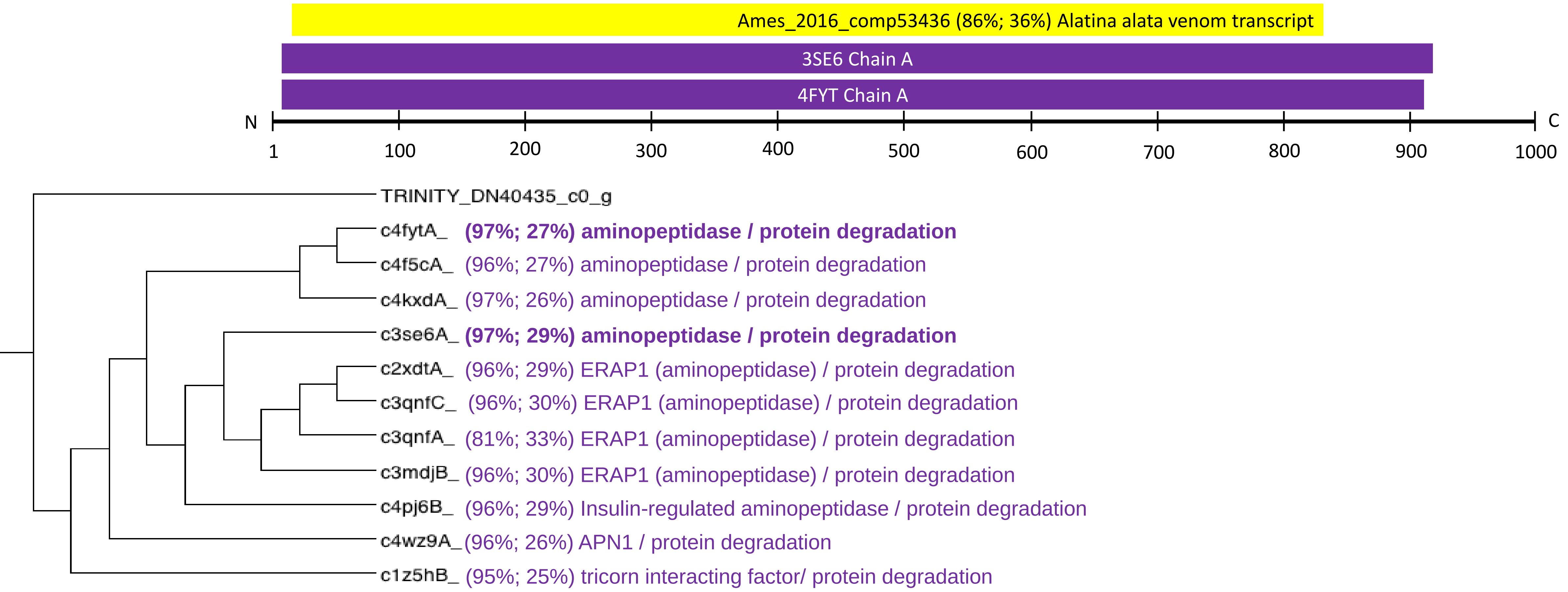

SeqID: TRINITY_DN21210_c1_g1_i1
Annotation: PC3-like endoprotease variant B
Identified by seq analysis: N

Key: (X%; Y%) A / B X = Alignment coverage Y= %ID between query and temple A = Molecule name B = Molecule description
NP= Alignment & %ID not provided by Phyre2; Green = same clade; Purple = Earlier common ancestor; Red = Non-valid entry; Blue = Not in tree

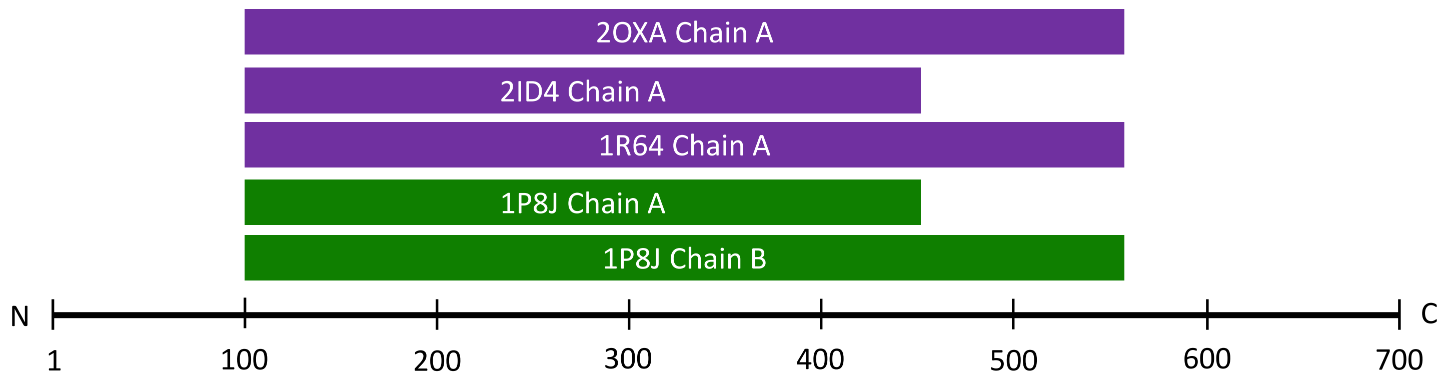

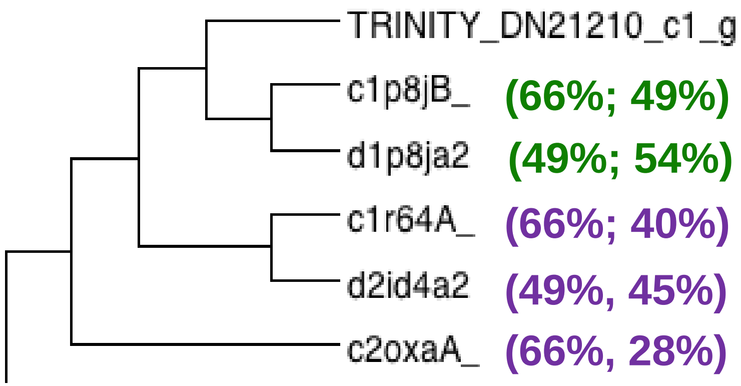

(66%; 49%) Furin/ calcium dependent serine endoprotease
(49%; 54%) Furin/ calcium dependent serine endoprotease
(66%; 40%) Kex2/ serine protease
(49%, 45%) Kex2/ serine protease
(66%, 28%) serine protease/shown to induce vascular leakage

SeqID: TRINITY_DN60273_c8_g1_i1
Annotation: peptidyl-prolyl cis-trans isomerase A
Identified by seq analysis: Y

Key: (X%; Y%) A / B X = Alignment coverage Y= %ID between query and temple A = Molecule name B = Molecule description
NP= Alignment & %ID not provided by Phyre2; Yellow = Sequence from database; Green = same clade; Purple = Earlier common ancestor; Red = Non-valid entry; Blue = Not in tree

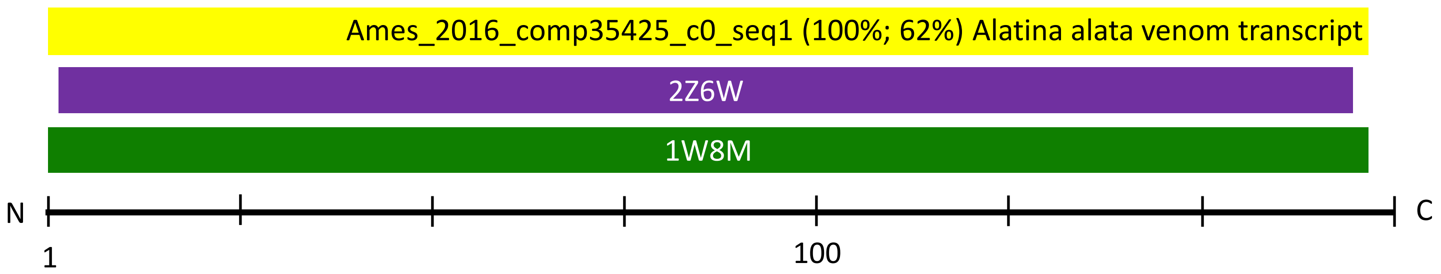

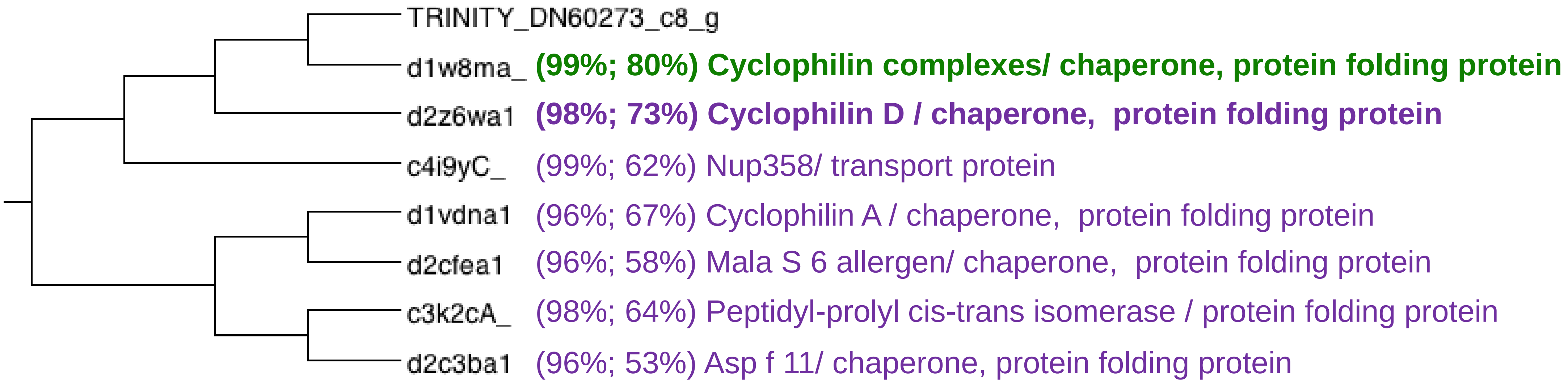

SeqID: TRINITY_DN6523_c1_g1_i1
Annotation: disulfide-isomerase A3
Identified by seq analysis: Y

Key: (X%; Y%) A / B X = Alignment coverage Y= %ID between query and temple A = Molecule name B = Molecule description
NP= Alignment & %ID not provided by Phyre2; Yellow = Sequence from database; Green = same clade; Purple = Earlier common ancestor; Red = Non-valid entry; Blue = Not in tree

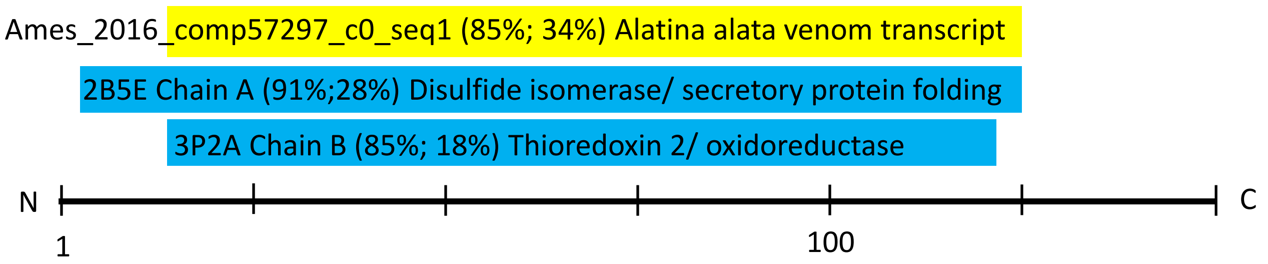

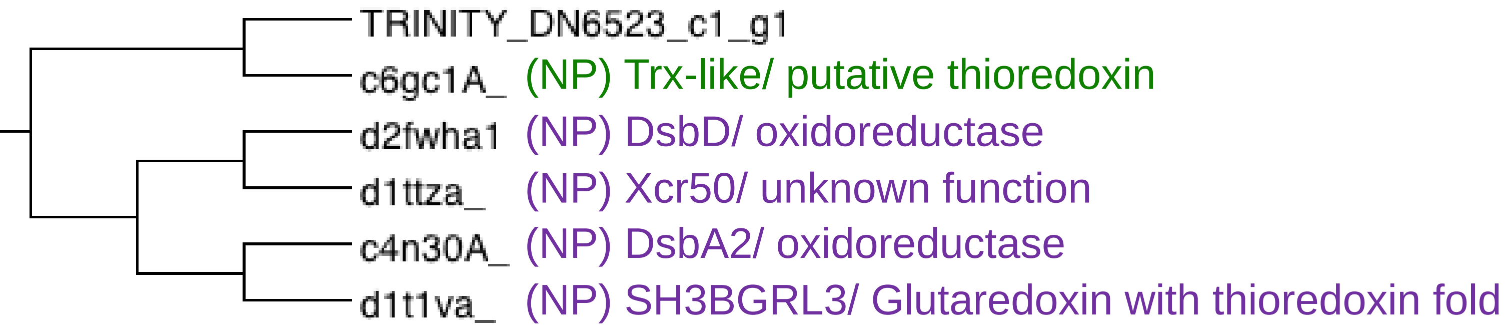

SeqID: TRINITY_DN58421_c12_g1_i1
Annotation: NA
Identified by seq analysis: N

Key: (X%; Y%) A / B X = Alignment coverage Y= %ID between query and temple A = Molecule name B = Molecule description
NP= Alignment & %ID not provided by Phyre2; Green = same clade; Purple = Earlier common ancestor; Red = Non-valid entry; Blue = Not in tree

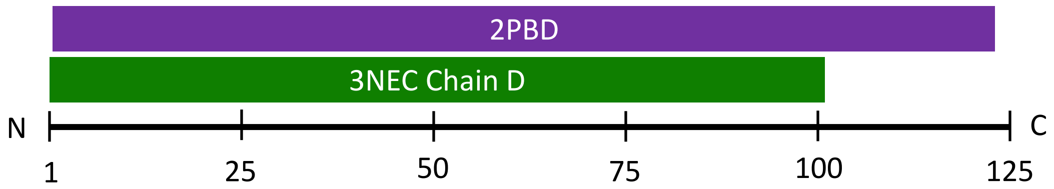

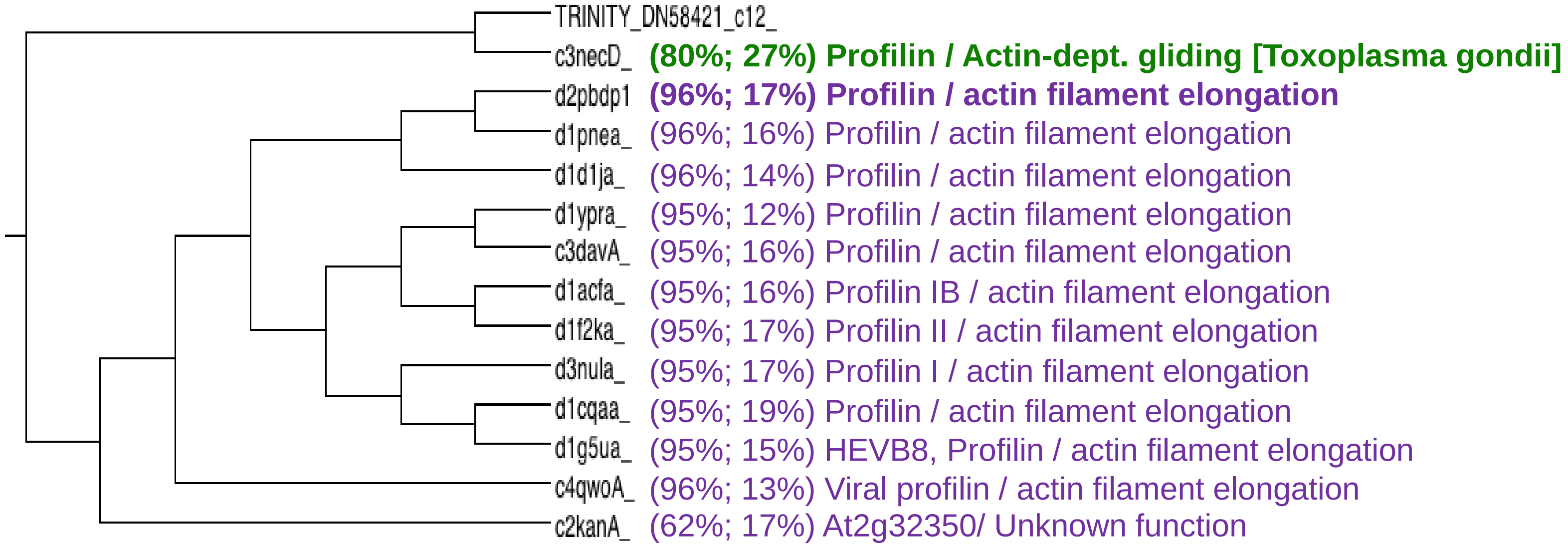

SeqID: TRINITY_DN10998_c2_g1_i1
Annotation: NA
Identified by seq analysis: N

Key: (X%; Y%) A / B X = Alignment coverage Y= %ID between query and temple A = Molecule name B = Molecule description
NP= Alignment & %ID not provided by Phyre2; Green = same clade; Purple = Earlier common ancestor; Red = Non-valid entry; Blue = Not in tree

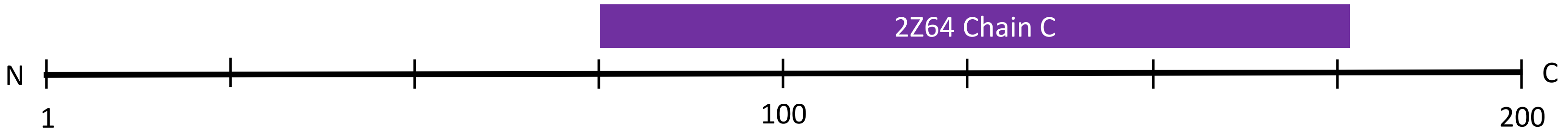

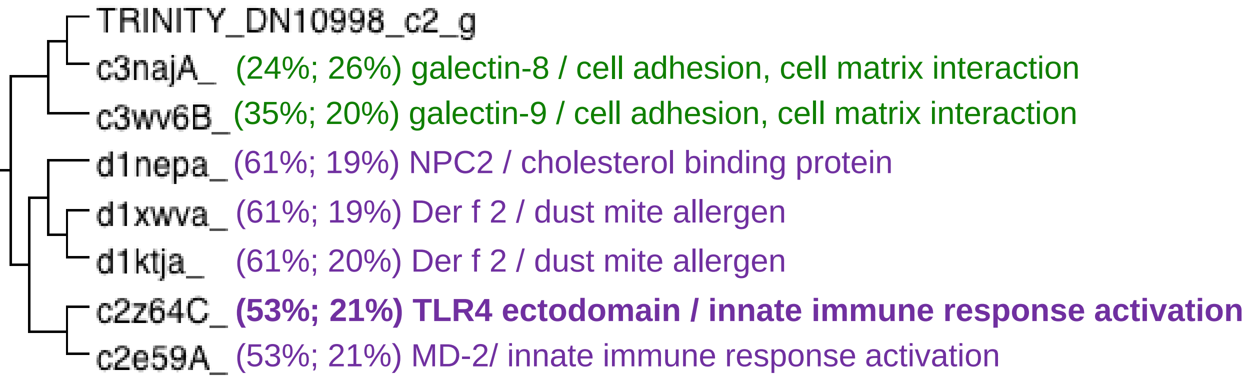

SeqID: TRINITY_DN57718_c2_g1_i1
Annotation: TKL Kinase
Identified by seq analysis: N

Key: (X%; Y%) A / B X = Alignment coverage Y= %ID between query and temple A = Molecule name B = Molecule description
NP= Alignment & %ID not provided by Phyre2; Green = same clade; Purple = Earlier common ancestor; Red = Non-valid entry; Blue = Not in tree

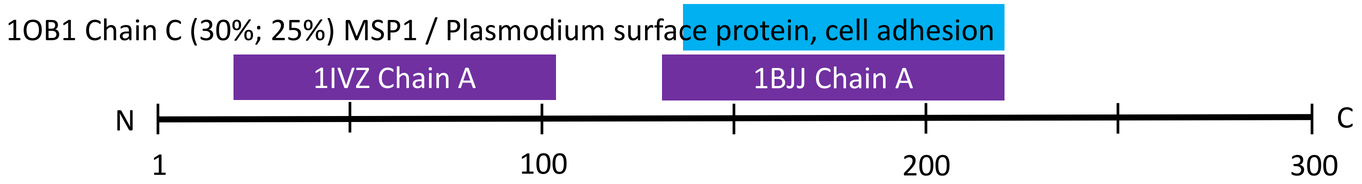

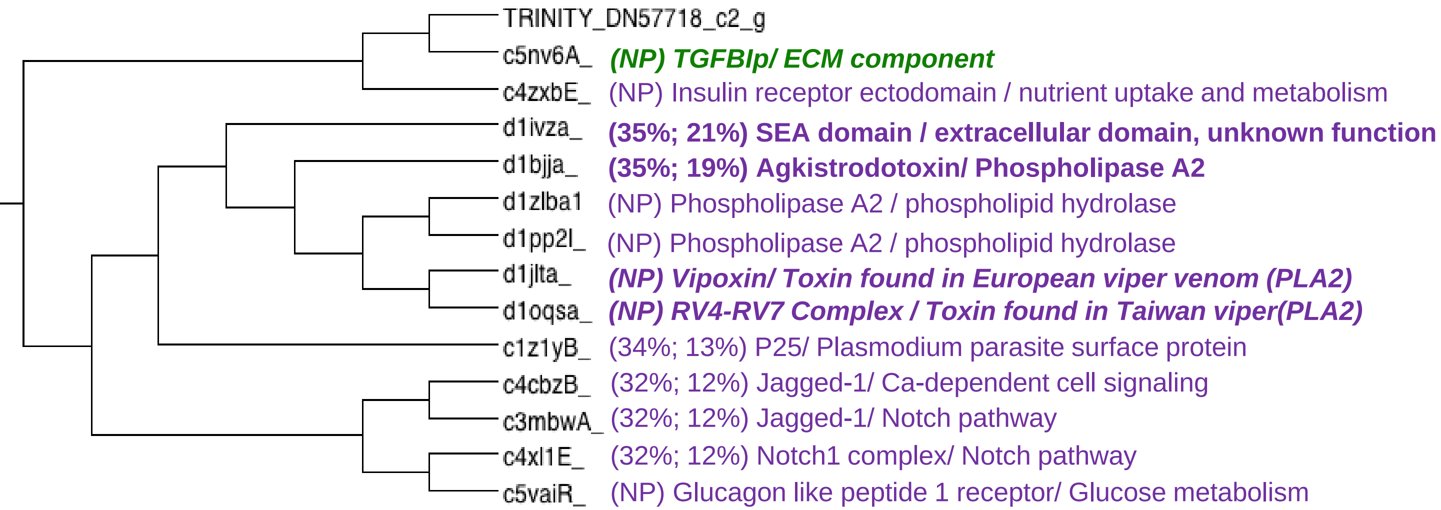

SeqID: TRINITY_DN60360_c0_g1_i1
Annotation: Nematoblast-specific nb012a
Identified by seq analysis: N

Key: (X%; Y%) A / B X = Alignment coverage Y= %ID between query and temple A = Molecule name B = Molecule description
NP= Alignment & %ID not provided by Phyre2; Green = same clade; Purple = Earlier common ancestor; Red = Non-valid entry; Blue = Not in tree

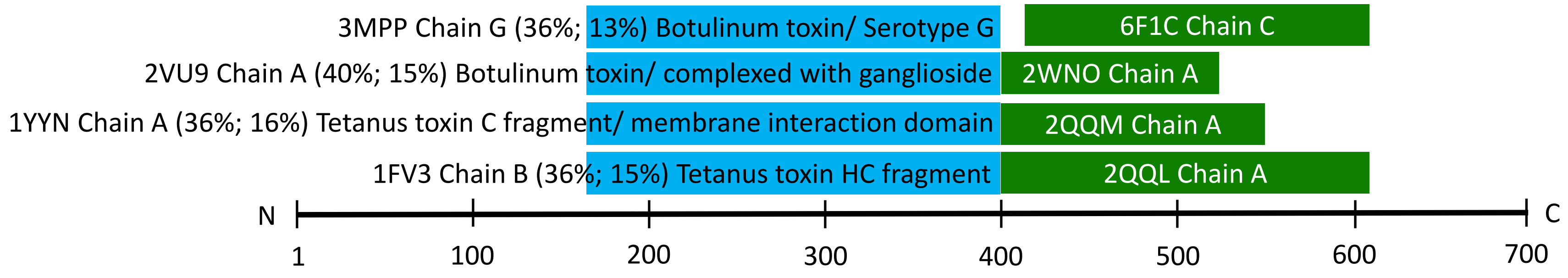

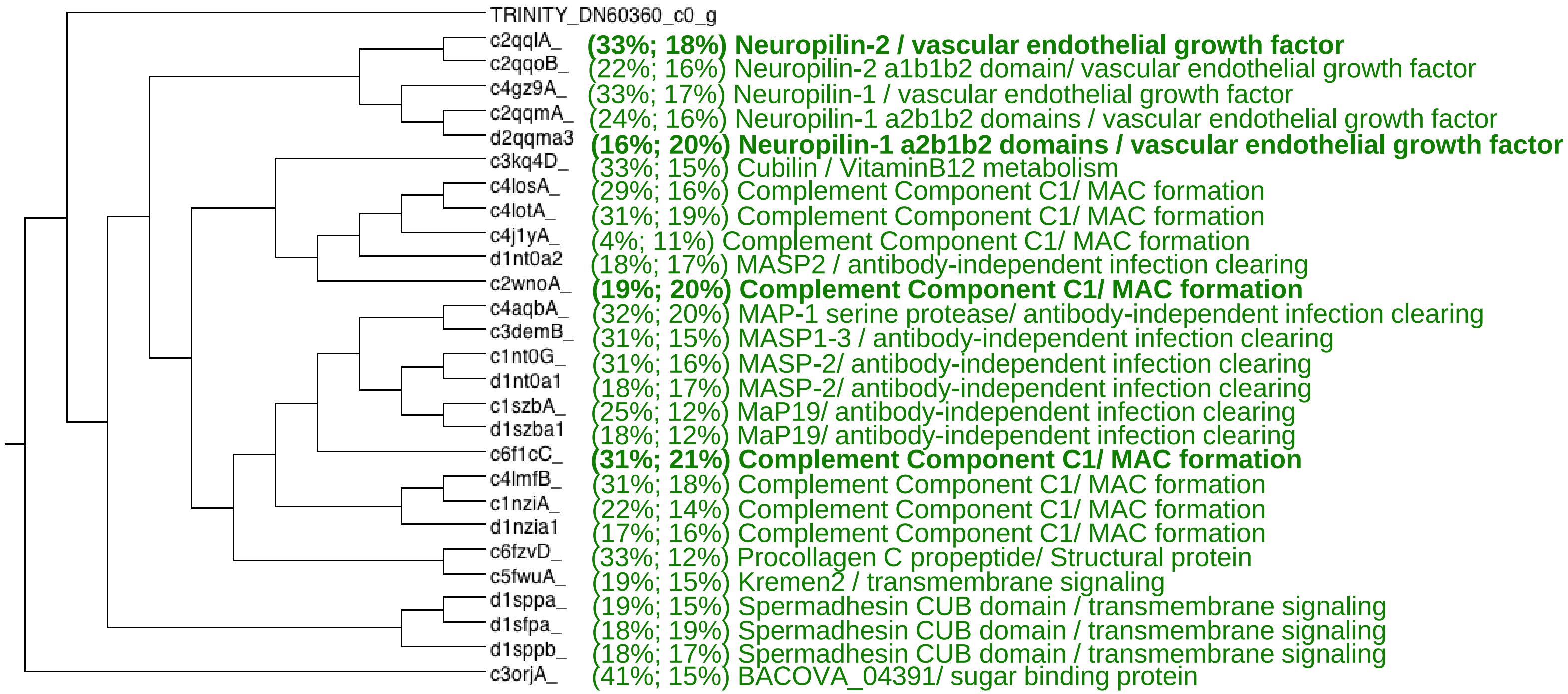

SeqID: TRINITY_DN3120_c1_g1_i1
Annotation: NA
Identified by seq analysis: N

Key: (X%; Y%) A / B X = Alignment coverage Y= %ID between query and temple A = Molecule name B = Molecule description
NP= Alignment & %ID not provided by Phyre2; Green = same clade; Purple = Earlier common ancestor; Red = Non-valid entry; Blue = Not in tree

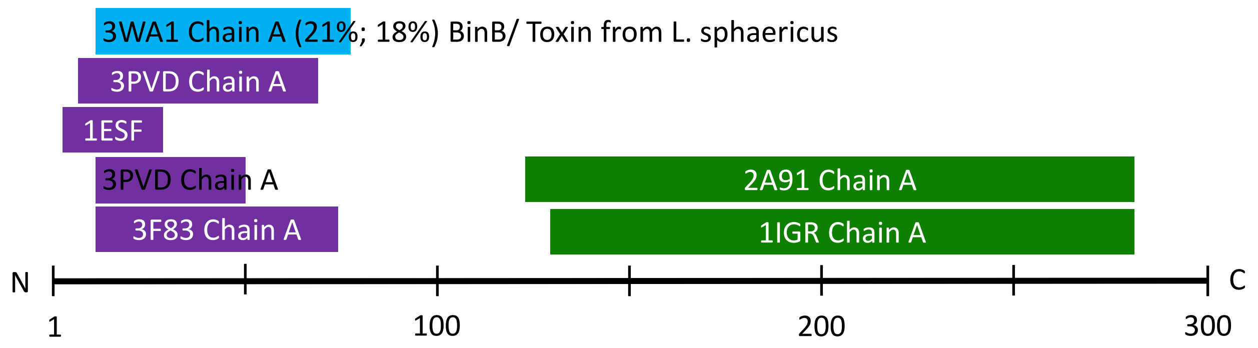

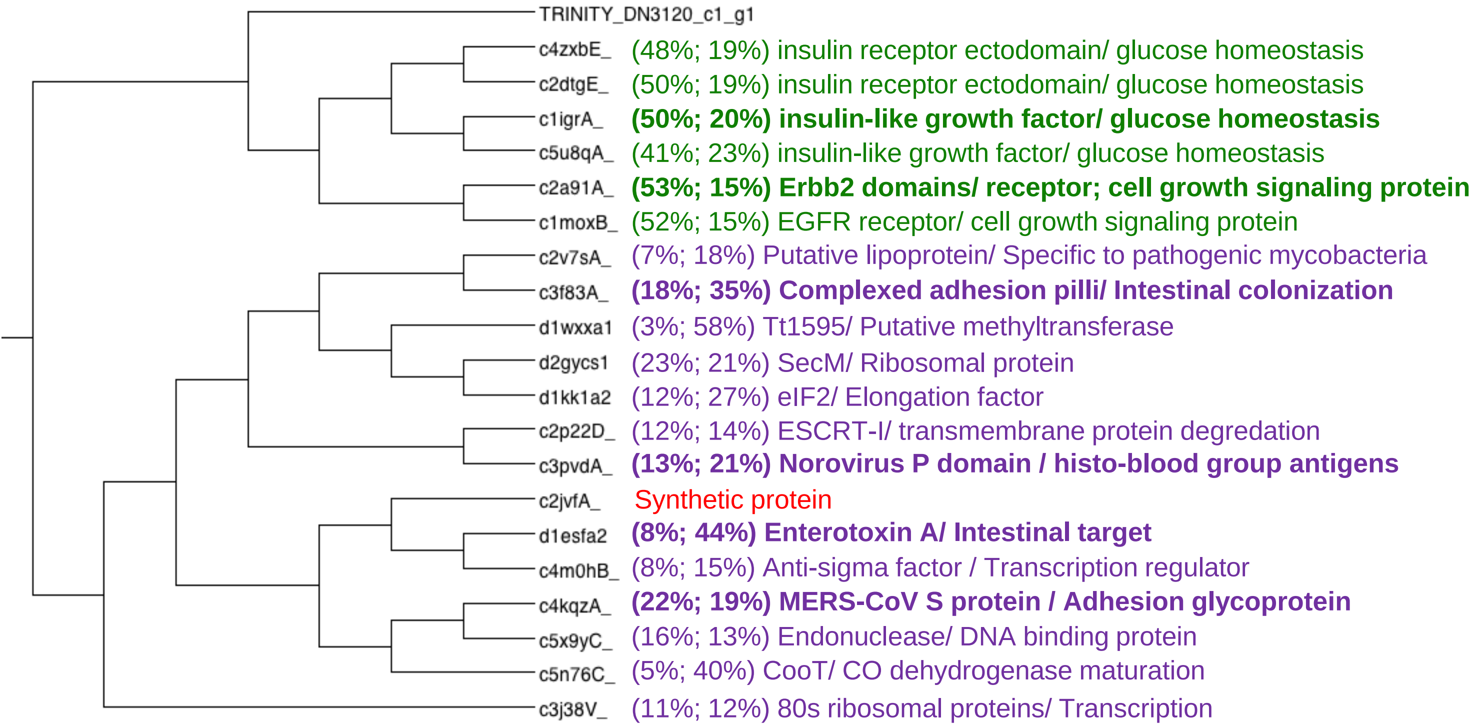

SeqID: TRINITY_DN52005_c0_g3_i2
Annotation: NA
Identified by seq analysis: N

Key: (X%; Y%) A / B X = Alignment coverage Y= %ID between query and temple A = Molecule name B = Molecule description
NP= Alignment & %ID not provided by Phyre2; Green = same clade; Purple = Earlier common ancestor; Red = Non-valid entry; Blue = Not in tree

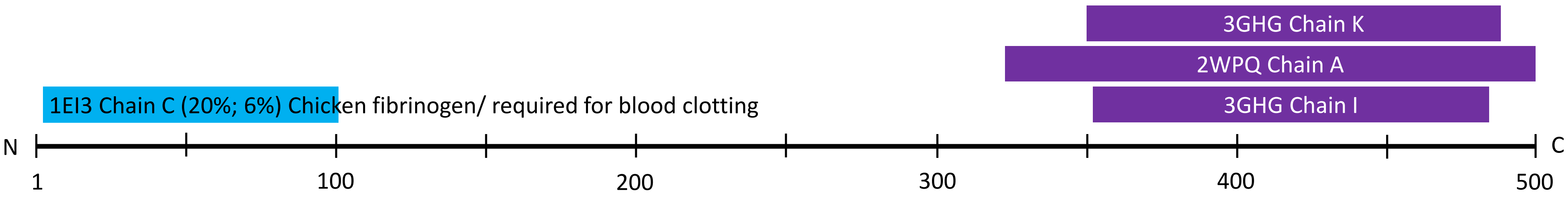

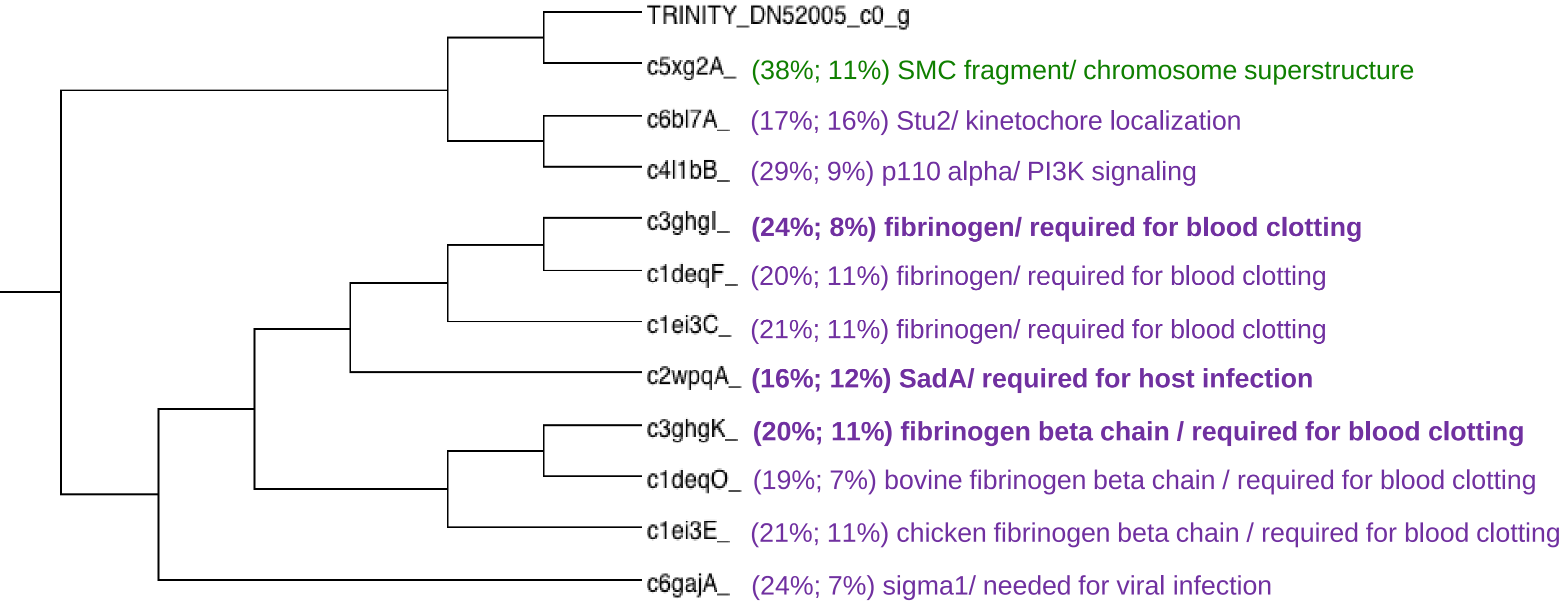

SeqID: TRINITY_DN3183_c1_g2_i1
Annotation: EGF-like repeat and discoidin I-like domain-containing 3
Identified by seq analysis: Y

Key: (X%; Y%) A / B X = Alignment coverage Y= %ID between query and temple A = Molecule name B = Molecule description
NP= Alignment & %ID not provided by Phyre2; Yellow = Sequence from database; Green = same clade; Purple = Earlier common ancestor; Red = Non-valid entry; Blue = Not in tree

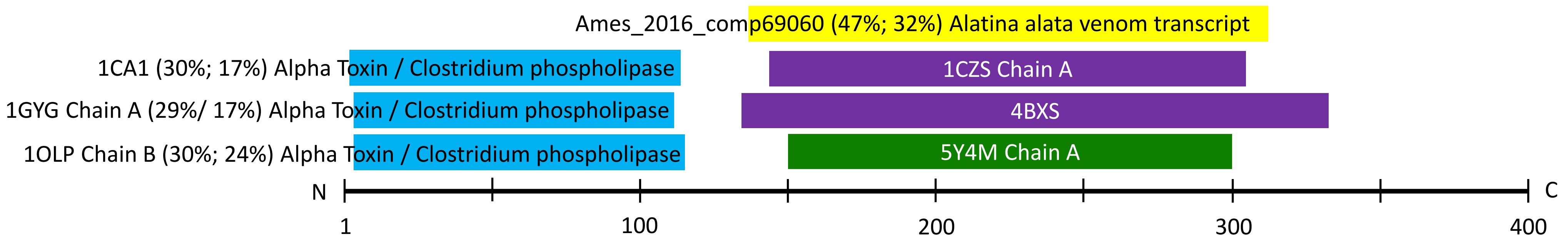

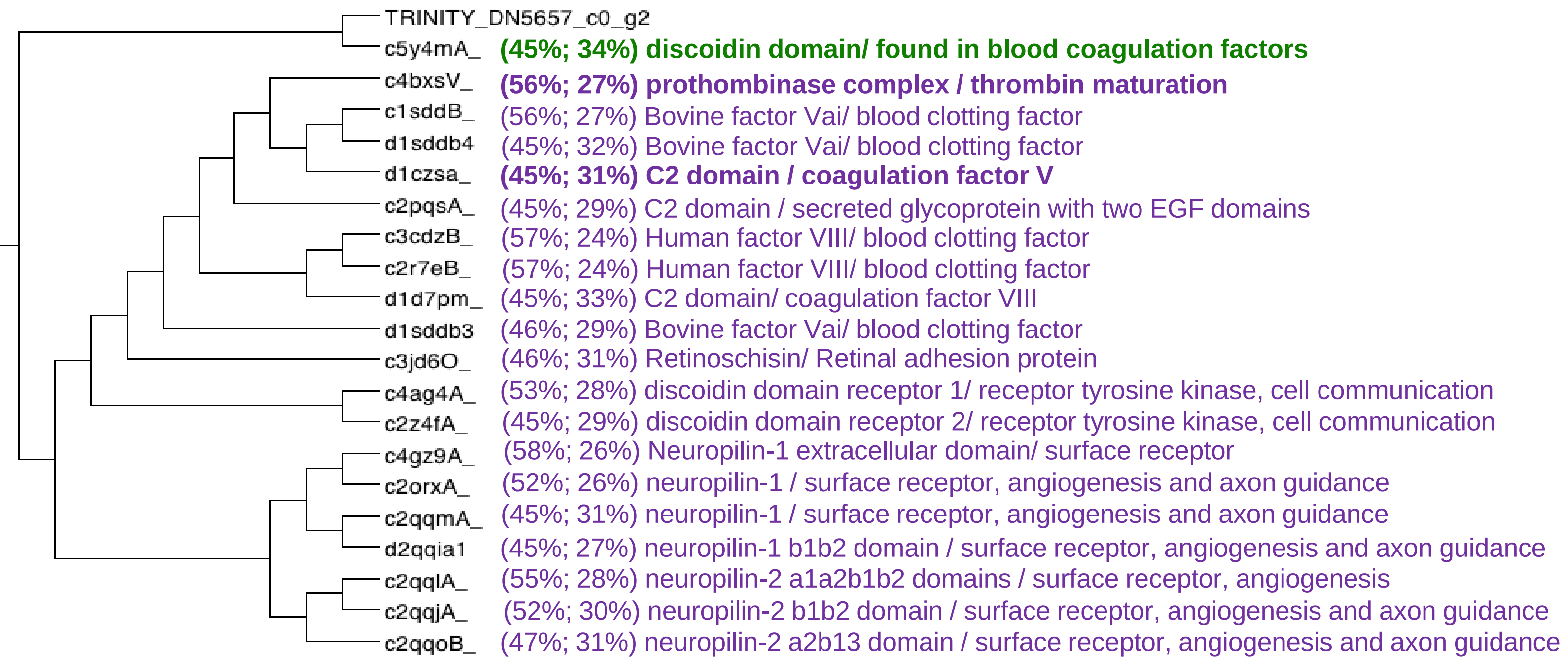

SeqID: TRINITY_DN9426_c0_g1_i2
Annotation: glutamine gamma-glutamyltransferase
Identified by seq analysis: N

Key: (X%; Y%) A / B   X = Alignment coverage   Y= %ID between query and temple   A = Molecule name   B = Molecule description
NP= Alignment & %ID not provided by Phyre2; Green = same clade; Purple = Earlier common ancestor; Red = Non-valid entry; Blue = Not in tree

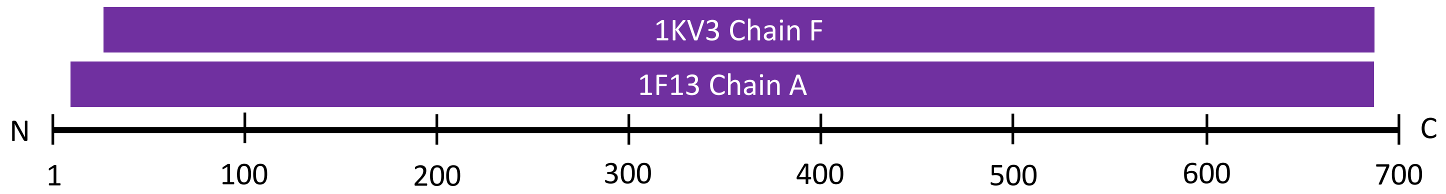

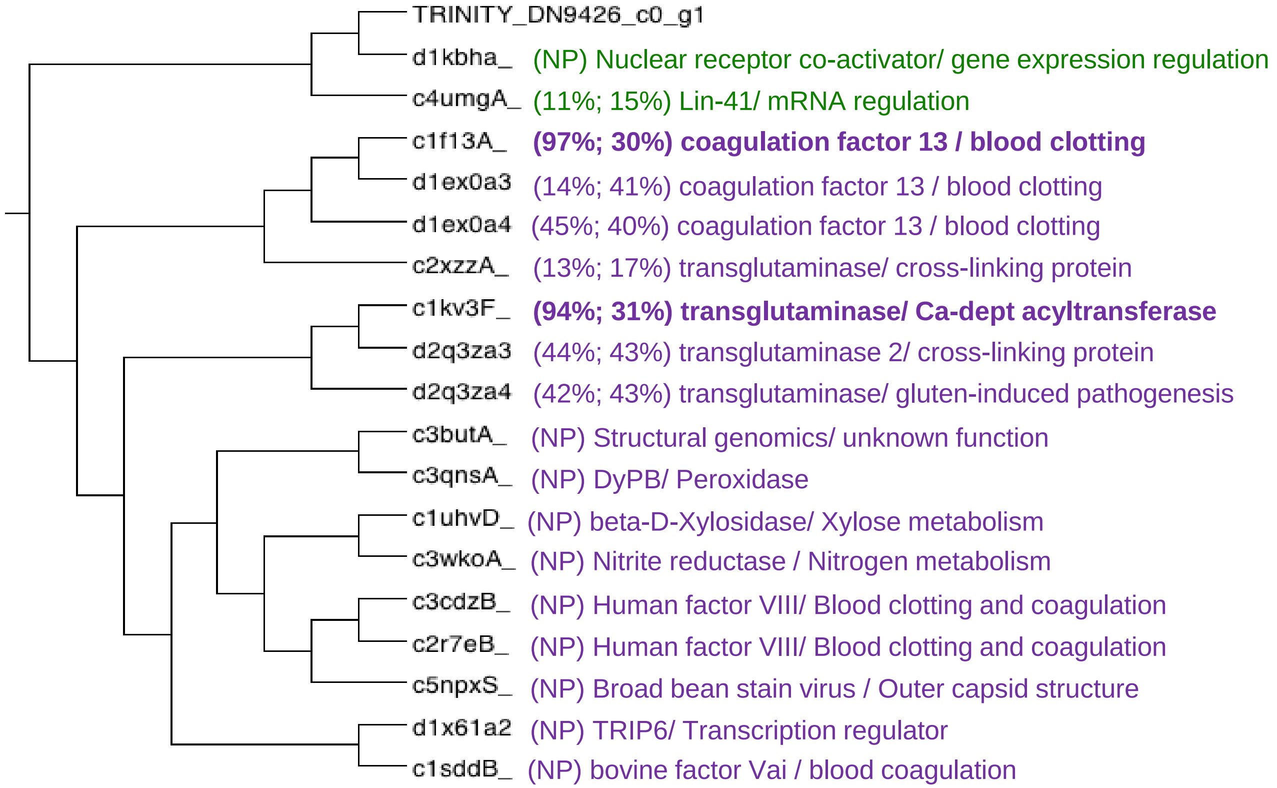

SeqID: TRINITY_DN3183_c0_g2_i1
Annotation: coagulation factor V-like
Identified by seq analysis: Y

Key: (X%; Y%) A / B X = Alignment coverage Y= %ID between query and temple A = Molecule name B = Molecule description
NP= Alignment & %ID not provided by Phyre2;
Yellow = Sequence from database; Green = same clade; Purple = Earlier common ancestor; Red = Non-valid entry; Blue = Not in tree

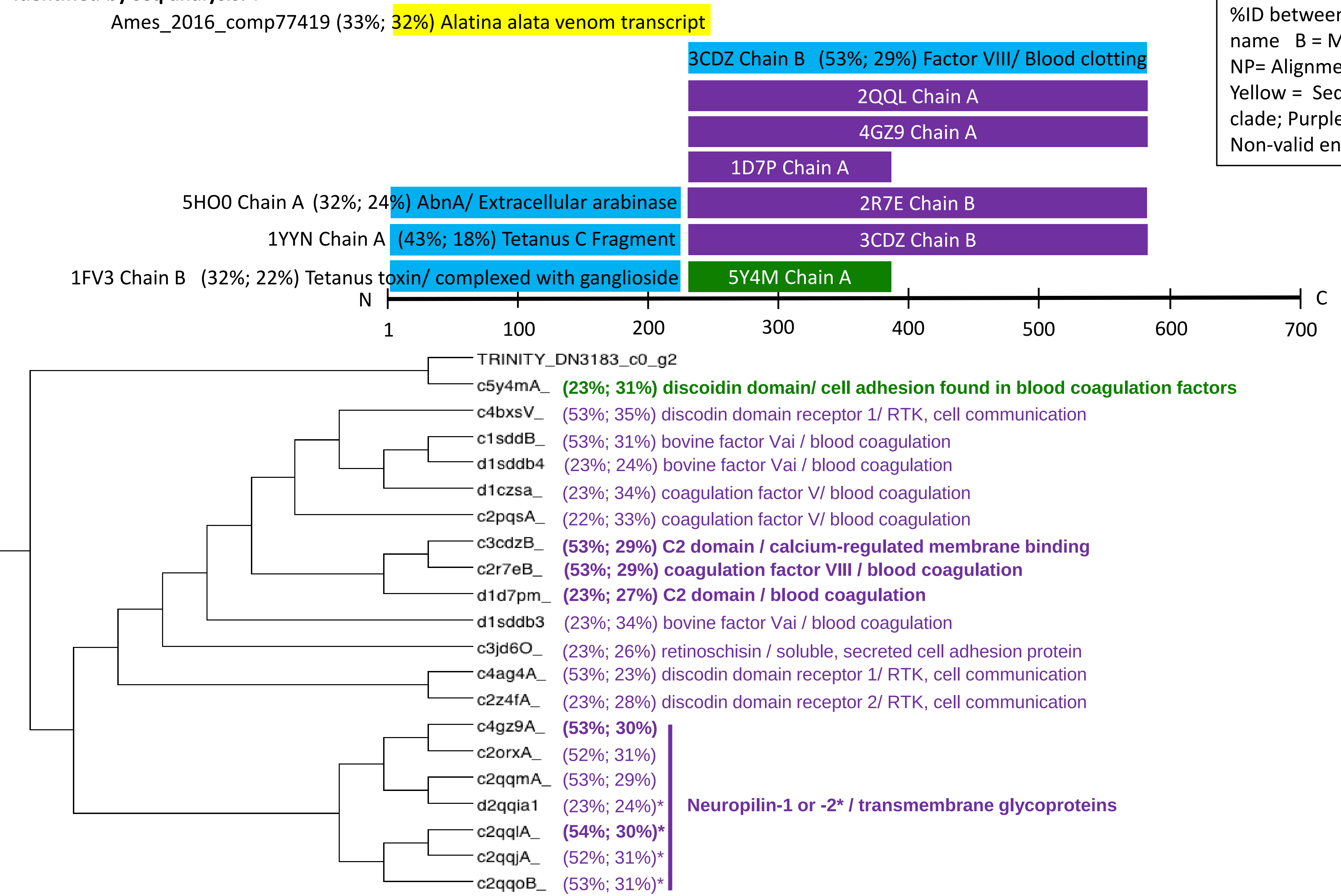

Supplement: Supplemental Information 2 — A: Domain alignment visualizations with representative sequences from major tree branches. B: Phylogeny made with DALI for structurally similar sequences, whose functions contained the keywords. Yellow = sequence from database; Green = same clade; Purple = earlier common ancestor; Red = non-valid entry; Blue = not in tree. [file peerj-09-12606-s002.pdf]
