# Supplementary material for: The cnidarian parasite Ceratonova shasta utilizes inherited and recruited venom-like compounds during infection
Source: PeerJ. 2021 Dec 15;9:e12606. doi: 10.7717/peerj.12606 (PMC8684318; doi:10.7717/peerj.12606)

“Inherited” venom-like compounds

TRINITY_DN4616_c0_g1
C-type Lectin

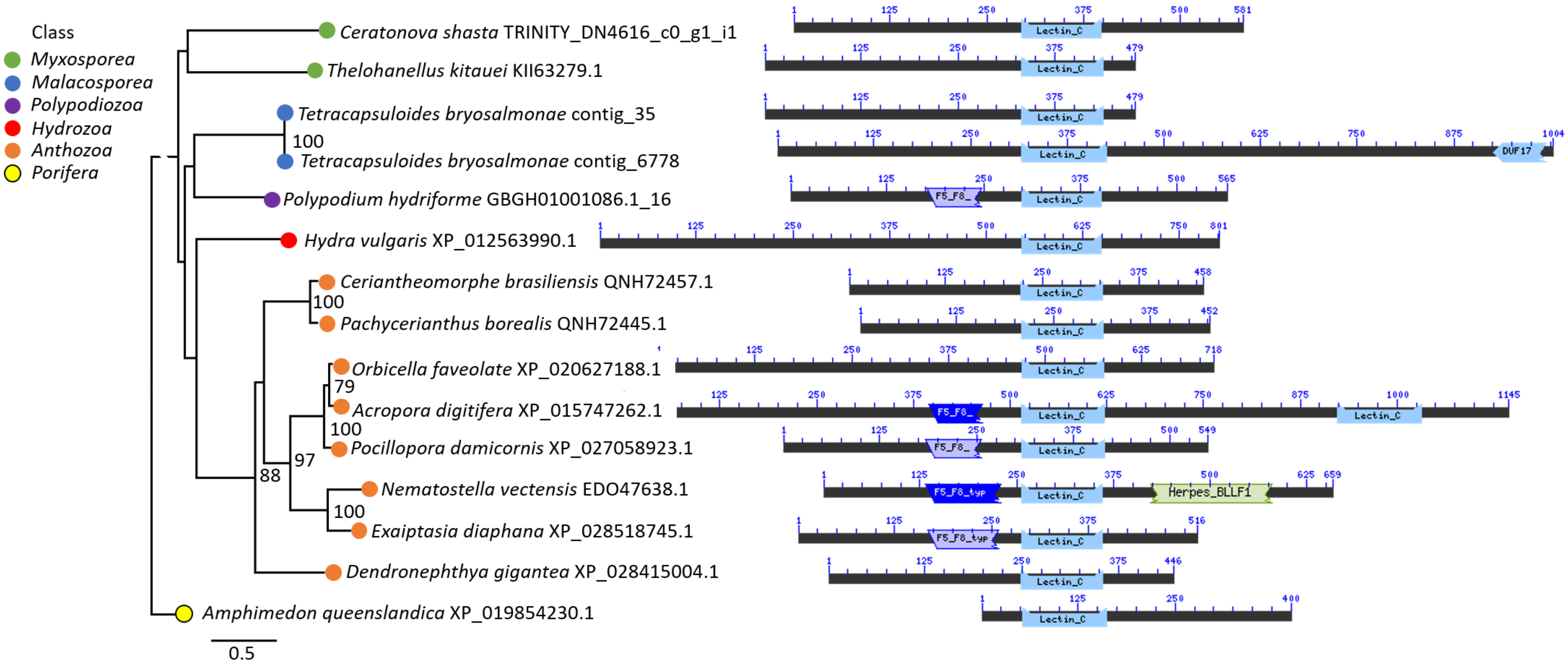

TRINITY_DN40342_c1_g1
Hyaluronidase

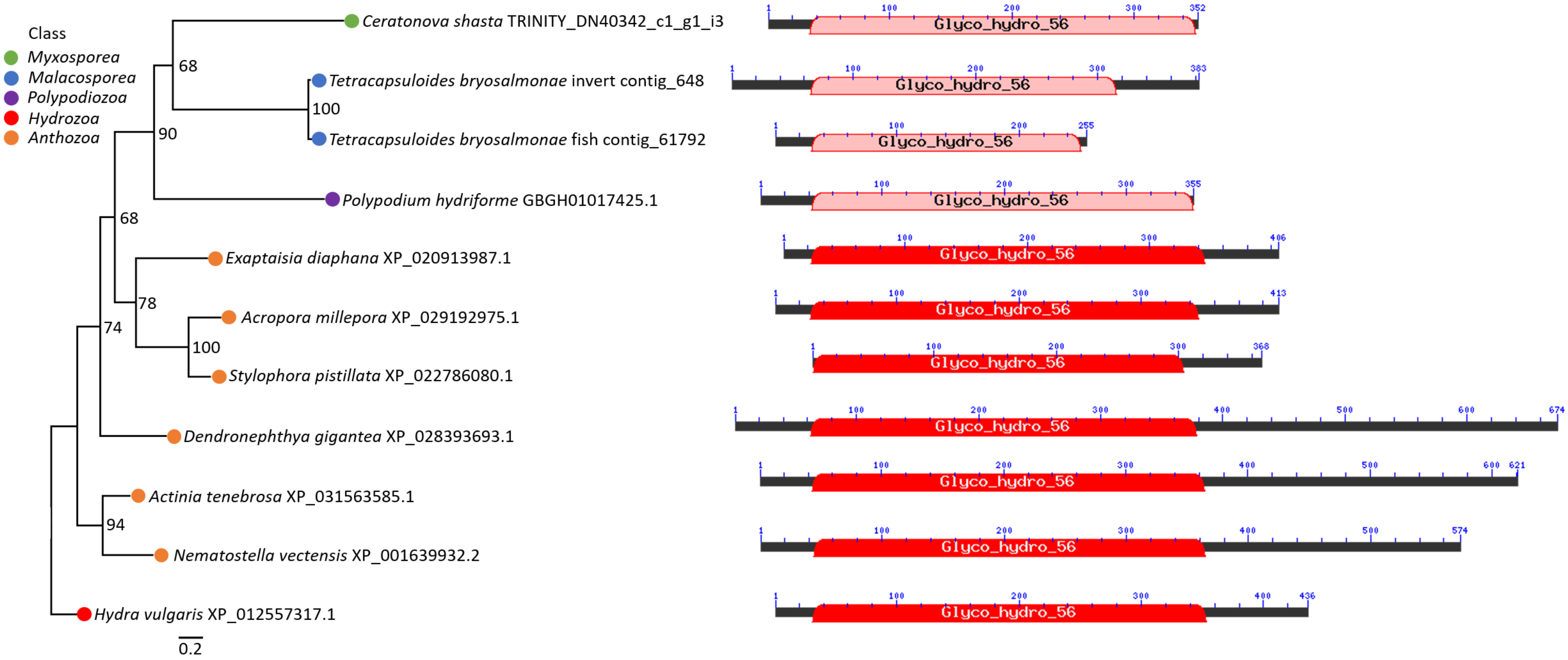

TRINITY_DN3183_c1_g2
Lactadherin

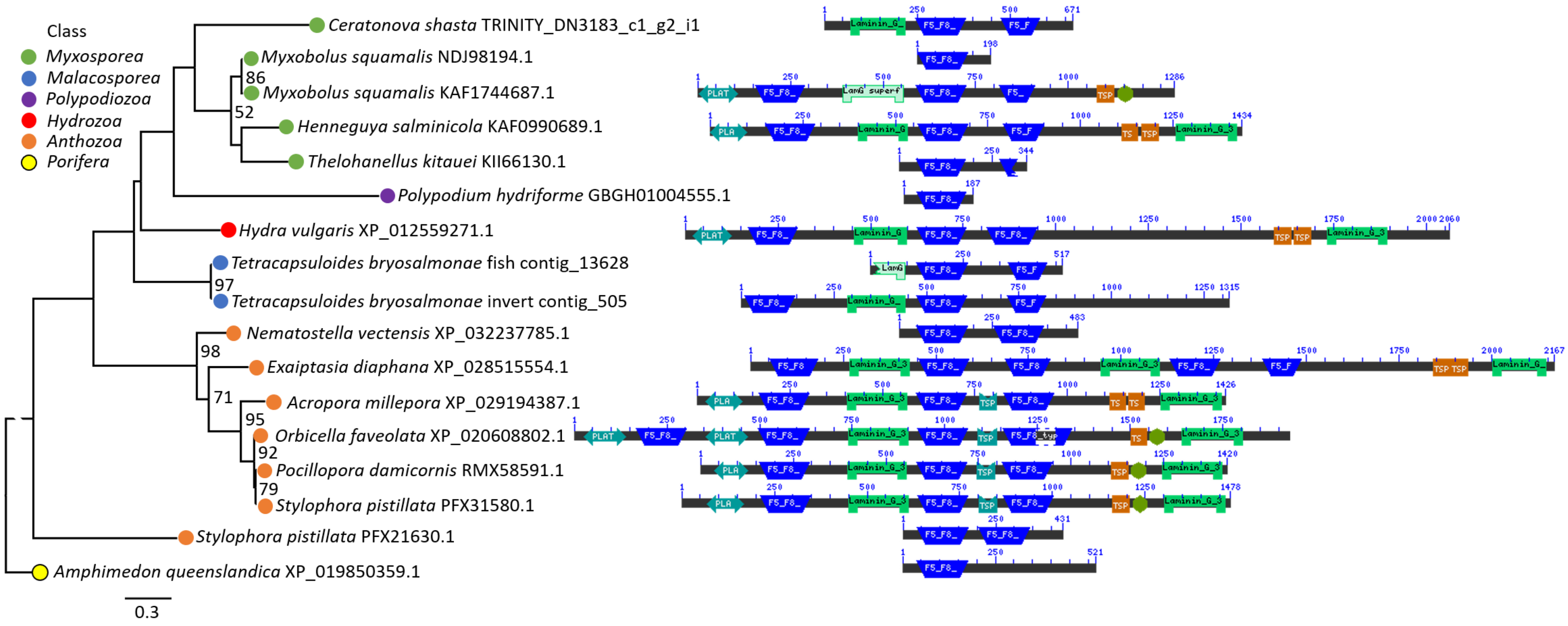

“Recruited” venom-like compounds

TRINITY_DN25741_c0_g1
Metallopeptidase

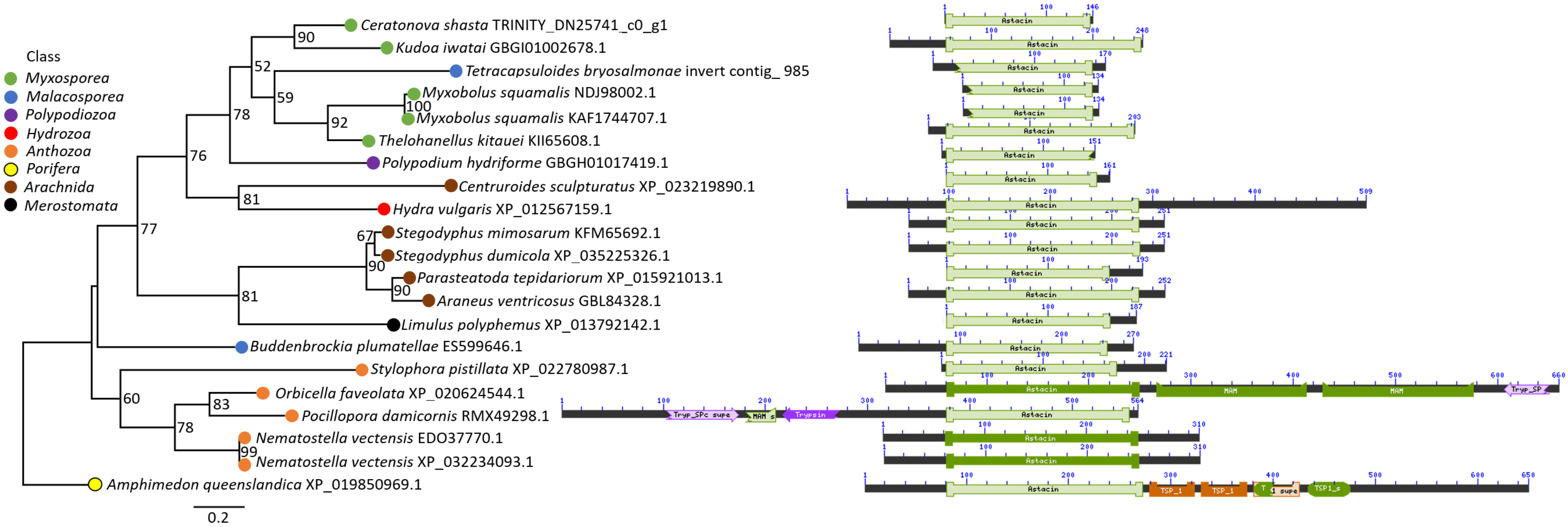

TRINITY_DN29246_c0_g1
Metallopeptidase

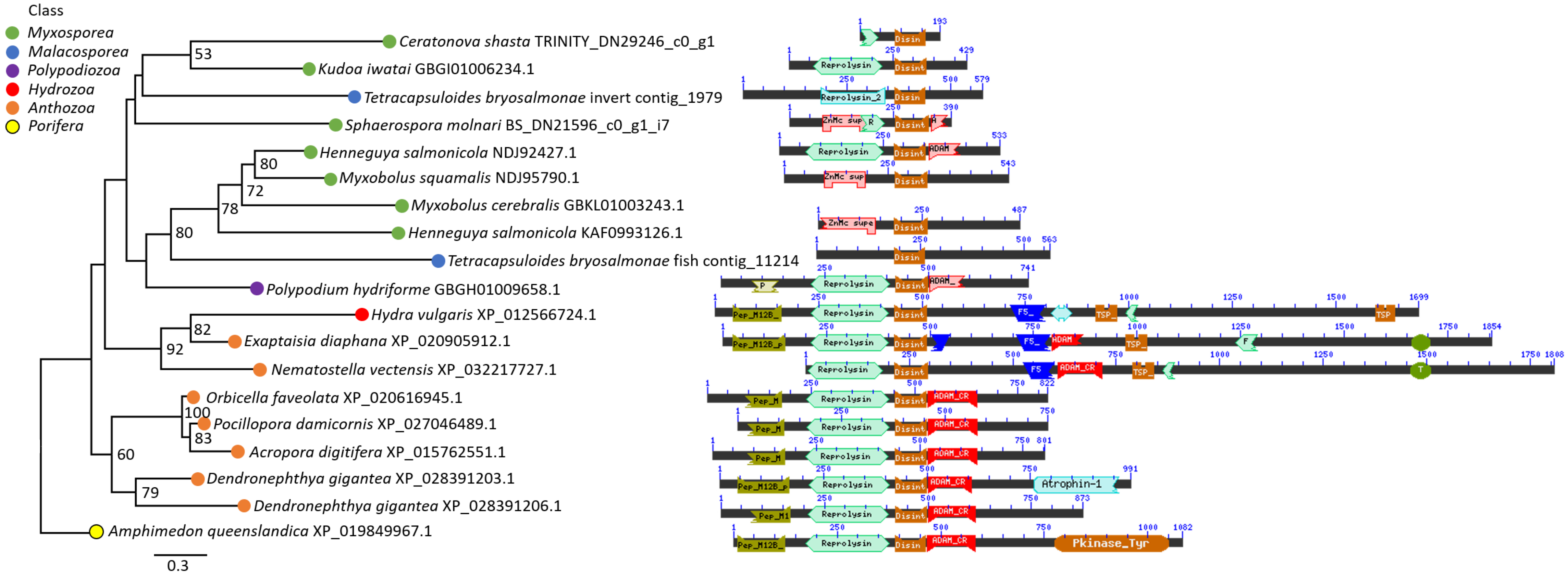

TRINITY_39886_c1_g1
Kunitz-type protein

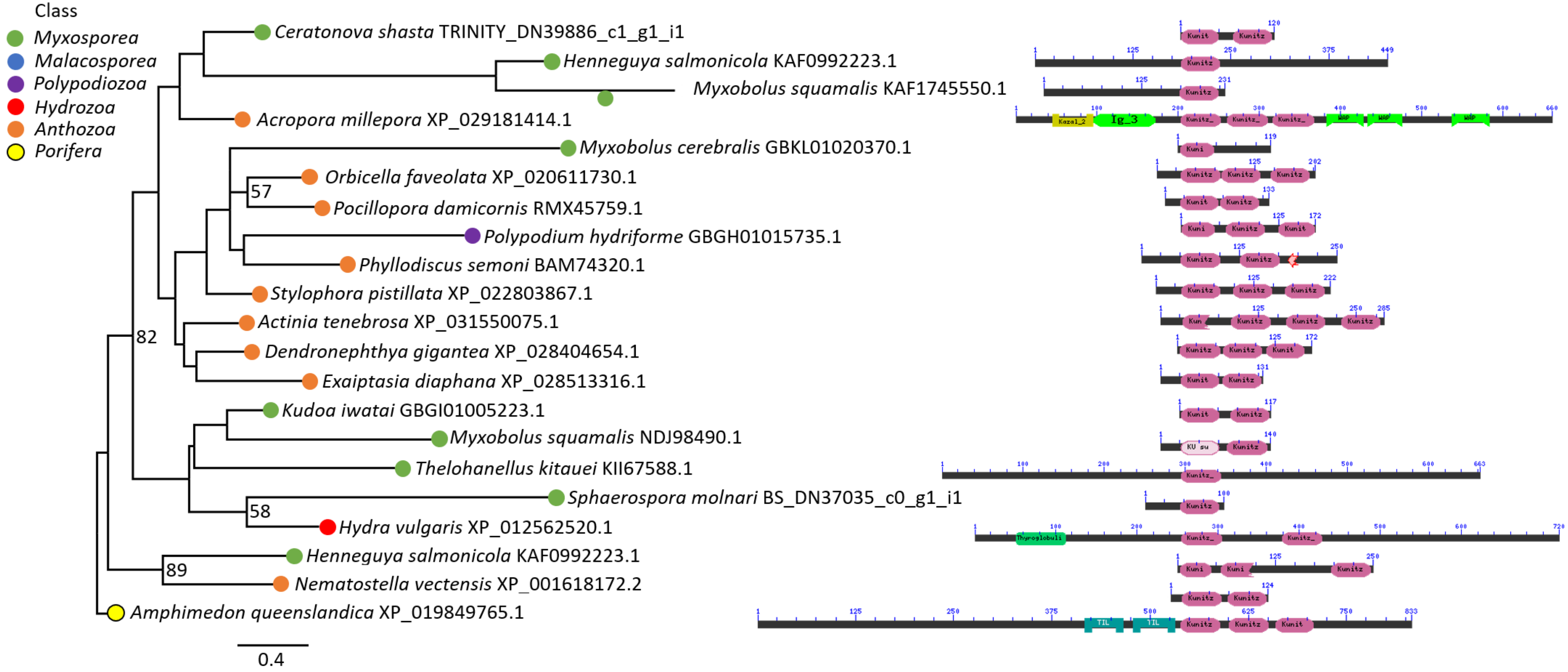

TRINITY_DN40098_c2_g1
Kunitz-type protein

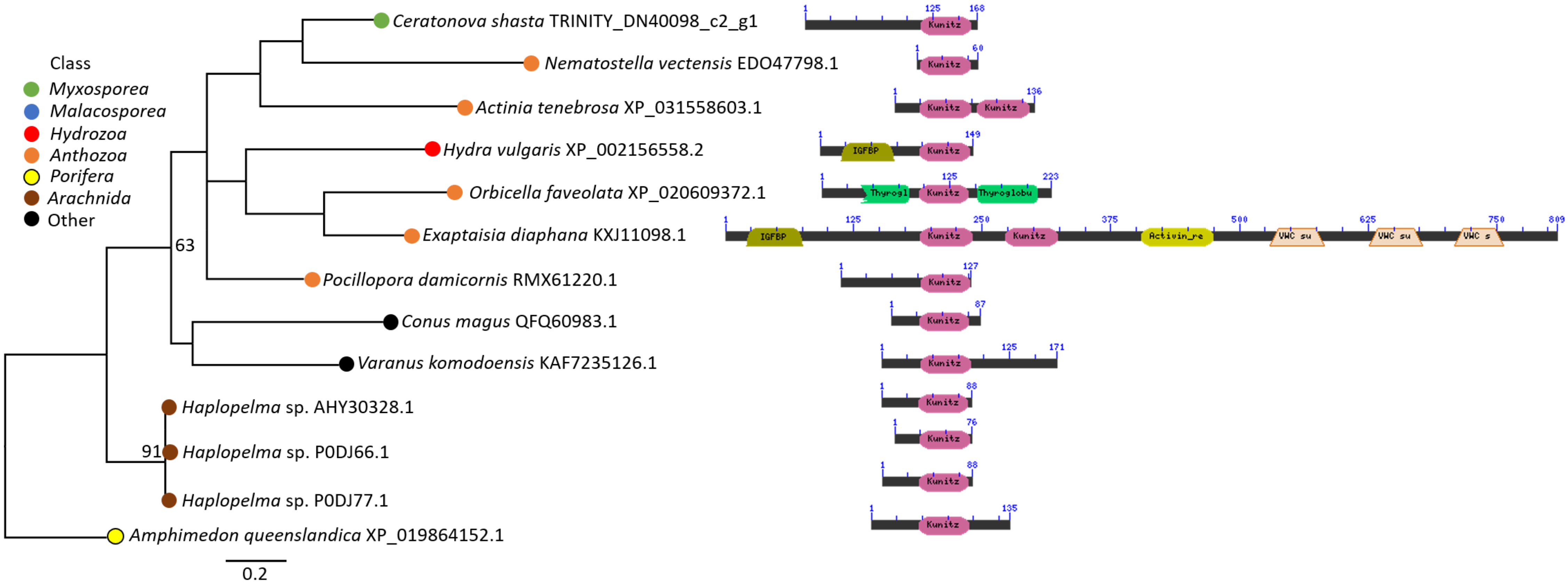

TRINITY_DN40387_c0_g2
Peptidase inhibitor 16

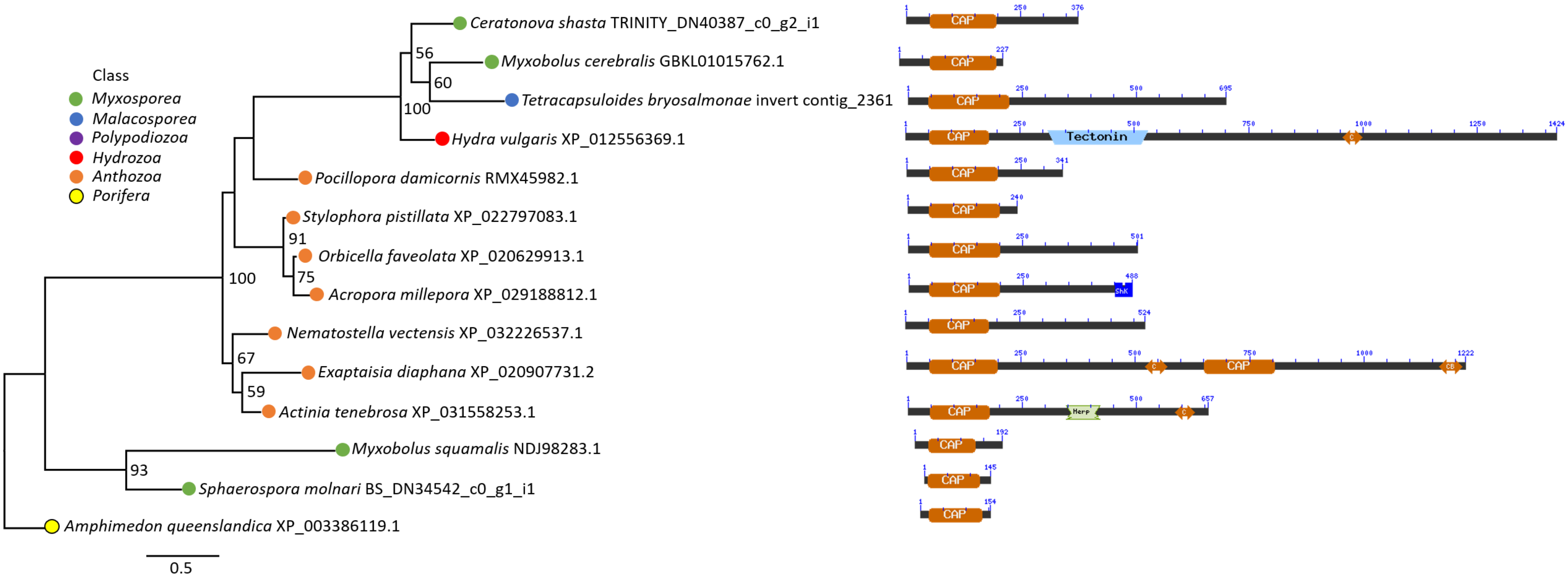

TRINITY_DN55967_c1_g1
Peptidase inhibitor 16

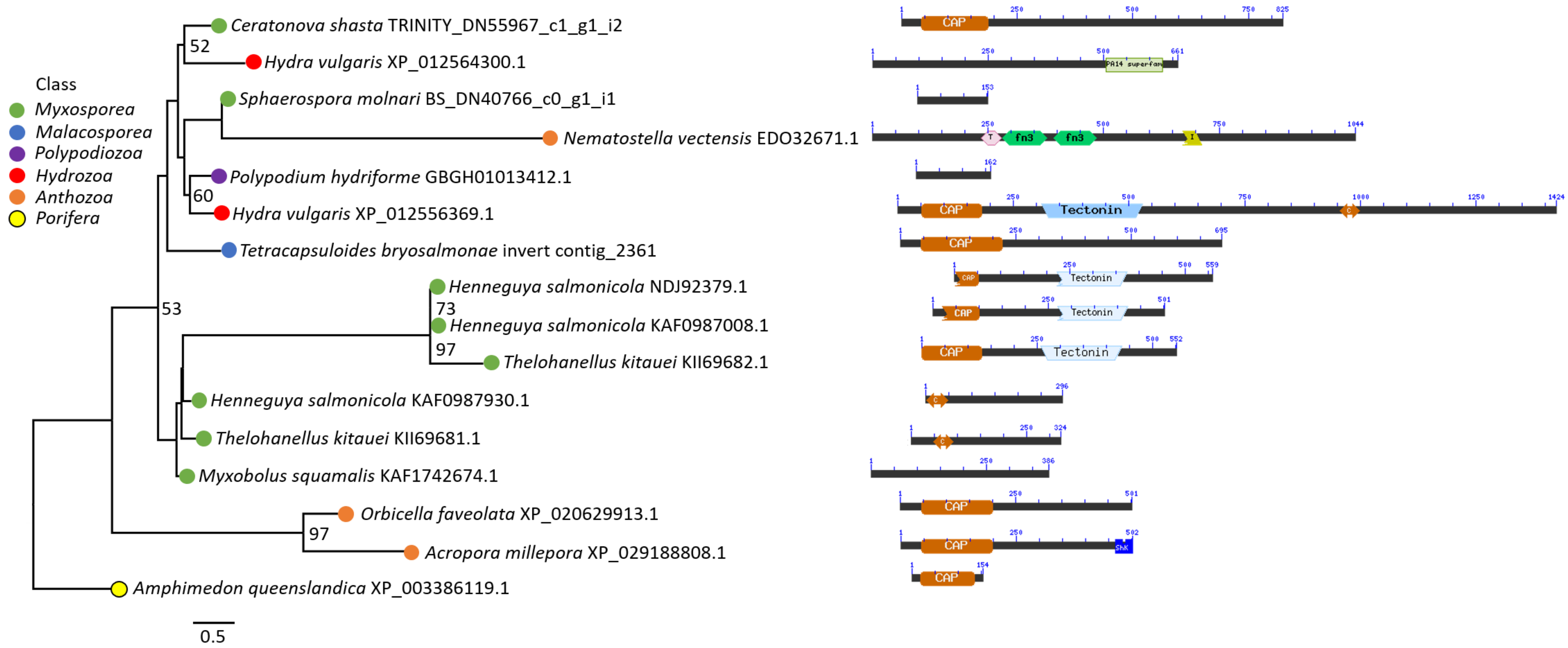

Supplement: Supplemental Information 3 — Phylogenies and protein domain alignments for nine venom-like compounds and most closely related sequences. Protein domains were annotated using the NCBI conserved domains search tool. [file peerj-09-12606-s003.pdf]
